# A Nanostrategy for Efficient Imaging‐Guided Antitumor Therapy through a Stimuli‐Responsive Branched Polymeric Prodrug

**DOI:** 10.1002/advs.201903243

**Published:** 2020-01-31

**Authors:** Hao Cai, Xinghang Dai, Xiaoming Wang, Ping Tan, Lei Gu, Qiang Luo, Xiuli Zheng, Zhiqian Li, Hongyan Zhu, Hu Zhang, Zhongwei Gu, Qiyong Gong, Kui Luo

**Affiliations:** ^1^ Huaxi MR Research Center (HMRRC) Department of Radiology Functional and Molecular Imaging Key Laboratory of Sichuan Province National Clinical Research Center for Geriatrics State Key Laboratory of Biotherapy West China Hospital Sichuan University Chengdu 610041 China; ^2^ West China School of Medicine Sichuan University Chengdu 610041 China; ^3^ Amgen Bioprocessing Centre Keck Graduate Institute Claremont CA 91711 USA; ^4^ National Engineering Research Center for Biomaterials Sichuan University Chengdu 610064 China

**Keywords:** biodegradability, branched polymers, drug delivery, stimuli‐responsive, theranostic nanomedicines

## Abstract

A stimuli‐responsive polymeric prodrug‐based nanotheranostic system with imaging agents (cyanine5.5 and gadolinium‐chelates) and a therapeutic agent paclitaxel (PTX) is prepared via polymerization and conjugating chemistry. The branched polymeric PTX‐Gd‐based nanoparticles (BP‐PTX‐Gd NPs) demonstrate excellent biocompatibility, and high stability under physiological conditions, but they stimuli‐responsively degrade and release PTX rapidly in a tumor microenvironment. The in vitro behavior of NPs labeled with fluorescent dyes is effectively monitored, and the NPs display high cytotoxicity to 4T1 cells similar to free PTX by impairing the function of microtubules, downregulating anti‐apoptotic protein Bcl‐2, and upregulating the expression of Bax, cleaved caspase‐3, cleaved caspase‐9, cleaved‐PARP, and p53 proteins. Great improvement in magnetic resonance imaging (MRI) is demonstrated by these NPs, and MRI accurately maps the temporal change profile of the tumor volume after injection of NPs and the tumor treatment process is also closely correlated with the *T*_1_ values measured from MRI, demonstrating the capability of providing real‐time feedback to the chemotherapeutic treatment effectiveness. The imaging‐guided chemotherapy to the 4T1 tumor in the mice model achieves an excellent anti‐tumor effect. This stimuli‐responsive polymeric nano‐agent opens a new door for efficient breast cancer treatment under the guidance of fluorescence/MRI.

## Graphical abstract

This study demonstrates a strategy to fabricate stimuli‐responsive branched polymeric prodrug‐based theranostic nanomedicine. The anticancer mechanism is studied well and the in vitro and vivo behaviors including biodistribution, retention, and anticancer efficacy can be monitored well by imaging. This stimuli‐responsive polymeric demonstrates great potential of a platform for cancer nanotheranostics.

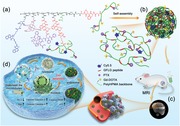

## 1 Introduction

Integration of diagnostic and therapeutic modalities within one polymeric drug delivery system has been attempted to be realized in theranostic nanomedicines.^1^ The nanomedicine will particularly play a role in the emerging era of personalized treatment by offering improved prognoses. Three key components are often required to formulate a theranostic nanomedicine: therapeutic drug, imaging agent, and their delivery vehicle.^2^

Delivery vehicles have been explored from a myriad of materials, including lipids, peptides, proteins, synthetic or naturally derived polymers, carbon, metals, and metal oxides.3g,h Synthetic polymers with different architectures, such as linear, star, branched, and dendritic shapes as well as supramolecular assemblies, have been employed for tumor specific diagnosis and therapy.^4^ Several formulations have received market approval.^5^ For example, hydrophobic anticancer drugs are conjugated to the poly[*N*‐(2‐hydroxypropyl) methacrylamide] (pHPMA) via a stimuli‐sensitive linker because pHPMA is nontoxic, nonimmunogenic, and nonbiodegradable during circulation.^6^ The pHPMA‐drug conjugates have improved water solubility of the drug, increased drug bioavailability, achieved controllable drug release, prolonged the half‐life of the conjugate in plasma, and enhanced accumulation of the drug in tumor tissues, leading to an improved antitumor efficacy and decreased inherent toxicity.^7^ Currently, several HPMA polymer‐drug conjugates are undergoing clinical trials, however, their low molecular weights (MWs) and short circulation time in vivo have hampered their clinical applications because the MWs must be controlled below the renal threshold (<50 kDa) to ensure biosafety of the polymer.^8^

Recently, biodegradable HPMA polymeric conjugates with MWs of 100–300 kDa have been prepared with enhanced accumulation in tumors and increased therapeutic indexes, while they still maintain excellent biosafety.^9^ In our previous studies, biodegradable high MWs branched pHPMA polymers have been demonstrated with high accumulation in tumors and excellent biosafety.^10^ Branched pHPMA polymers have exhibited special characteristics, such as self‐assembly properties, easy preparation, a unique topological structure, and more functional groups for conjugation.^11^ More functional groups allow covalent conjugation with multimodal agents, including therapeutic agents and imaging probes. In addition, these branched polymers are generally prepared via one‐pot reversible addition‐fragmentation chain transfer (RAFT) polymerization, so synthesis and purification of these polymers are ready for scale‐up manufacture. Although our previously reported branched pHPMA‐based drug delivery systems have shown enhanced anticancer activities compared to free drugs,[qv: 10,11b] improvements in the therapeutic indexes may be achieved via optimization of its molecular structure and conjugated agents.

The imaging modality has been exploited by specific molecular probes or contrast agents. Magnetic resonance imaging (MRI), one of the most commonly used medical diagnostic techniques, possesses unique features including noninvasiveness, no exposure to ionizing radiation, high contrast in soft tissues, and high spatiotemporal resolution.^12^ MRI‐based polymeric theranostics have been extensively studied including contrast agents and polymers, such as gadolinium‐tetraazacyclododecanetetraacetic acid (Gd‐DOTA), gadolinium‐diethylenetriamine penta‐acetic acid (Gd‐DTPA), polypeptides, pHPMA, polyamido‐amine dendrimers (pAMAMs), etc.^13^ Although pAMAMs conjugated with contrast agents have been observed with high relaxivity, efficient loading, and enhanced contrast, inherent toxicity of pAMAMs prevents further development of the dendrimer‐based conjugate.^14^ Optical imaging by covalently attaching fluorescent probes such as small fluorophores and fluorescent proteins to a polymer offers high image sensitivity, low bleaching rate, and low toxicity to cells, and living organisms, allowing effective evaluation of drug delivery process and intracellularly monitoring the tempospatial profile of the administrated drug.15b MRI provides macroscopic information with a submillimeter spatial resolution, and fluorescence imaging detects microcosmic details at a subcellular level.^16^ A combination of MRI and fluorescence imaging is a very promising multimodal imaging strategy for theranostics. Many elaborately designed systems have been reported for in vivo simultaneous MRI and fluorescence imaging.^17^ However, to our knowledge, there are currently very few biodegradable branched polymer‐based nanomedicines that combine multimodal imaging with tumor therapy for cancer research.

**Scheme 1:**
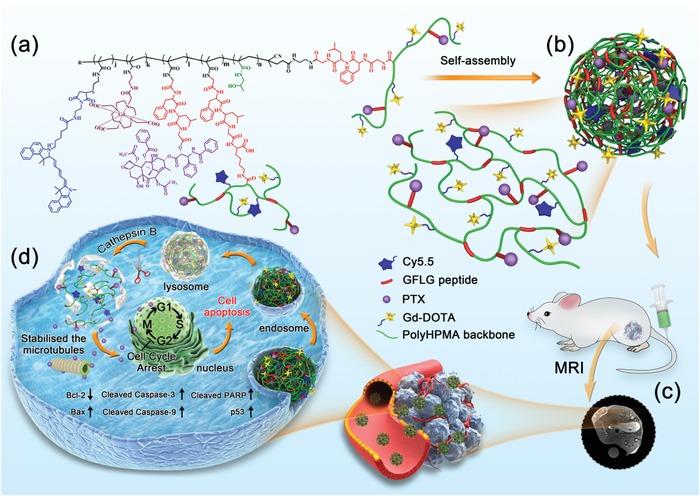
Schematic illustration of cathepsin B‐responsive biodegradable theranostic nanomedicine derived from branched pHPMA. a) Chemical structure of branched pHPMA‐PTX‐Gd conjugates. b) BP‐PTX‐Gd NPs formed through self‐assembly of the conjugate with hydrophilic and hydrophobic moieties. c) MR imaging in vivo of the delivery process of BP‐PTX‐Gd NPs to tumor sites via the EPR effect. d) BP‐PTX‐Gd NPs uptake and intracellular trafficking through lysosomal endocytosis pathways, and intracellular biodegradation in the overexpressed cathepsin B microenvironment and controlled drug release. Then, the released PTX inhibited cell mitosis by stabilizing microtubules and activated the corresponding apoptotic pathway to induce apoptosis.

Herein, we proposed to construct a novel biodegradable multifunctional theranostic nanoplatform based on branched polymers for simultaneous diagnosis and treatment of breast cancer. By integrating a Gly‐Phe‐Leu‐Gly (GFLG) tetrapeptide, an anticancer drug PTX, a fluorescence dye cyanine5.5 (Cy5.5), and an MRI contrast agent Gd‐DOTA into a branched pHPMA polymer through RAFT polymerization, click chemistry, and chemical complexation (**Scheme** **1**a) , the as‐prepared product would self‐assemble into a theranostic nanomedicine (BP‐PTX‐Gd NPs) (Scheme 1b). After the nanomedicine was uptaken by tumor cells, it would be capable of offering both MRI and fluorescence imaging (Scheme 1c) and releasing the anticancer drug for chemotherapy (Scheme 1d). The polymer backbone would be degraded into small fragments in the presence of overexpressed cathepsin B in the tumor cellular microenvironment (Scheme 1d) and the small fragments would be cleared to avoid toxic buildup of the delivery vector and the MRI contrast agent in the body. This cathepsin B‐responsive biodegradable theranostic nanomedicine derived from the branched pHPMA polymer could be formulated with other anticancer drugs for a variety of diseases.

## 2 Results and Discussion

### 2.1 Preparation and Characterization of BP‐PTX‐Gd NPs

The proposed theranostic nanomedicine for simultaneous diagnosis and treatment was prepared with the detailed synthesis route shown in Figure S1 in the Supporting Information: the branched polymer backbone was synthesized by the one‐pot method using RAFT polymerization using enzyme‐responsive chain transfer agents; the anticancer drug PTX was loaded onto the branched polymer via an enzyme‐responsive linker; Cy5.5 for fluorescence imaging was attached to the polymer chain via thiol‐ene click reaction and the MRI contrast agent Gd(III) chelated in DOTA was covalently bound to the polymer side chain. The GFLG peptide‐containing degradable dimethacrylate monomer, MA‐GFLGK‐MA, was used as a crosslinker. MA‐GFLG‐CTA simultaneously acted as a chain transfer agent (CTA) and a monomer due to the existence of two functional groups: a dithiobenzoate group and a methacrylate group. Therefore, the degradable drug‐loaded branched pHPMA was obtained from one‐step reaction. After purification, the dithiopyridine group in the polymer was removed by dithiothreitol (DTT) to reveal the thiol groups for covalently binding to the maleimide Cy5.5 through a highly efficient thiol‐ene click reaction. Finally, the obtained Cy5.5‐labeled polymer was chelated with GdCl_3_·6H_2_O to yield the proposed product.

**Figure 1:**
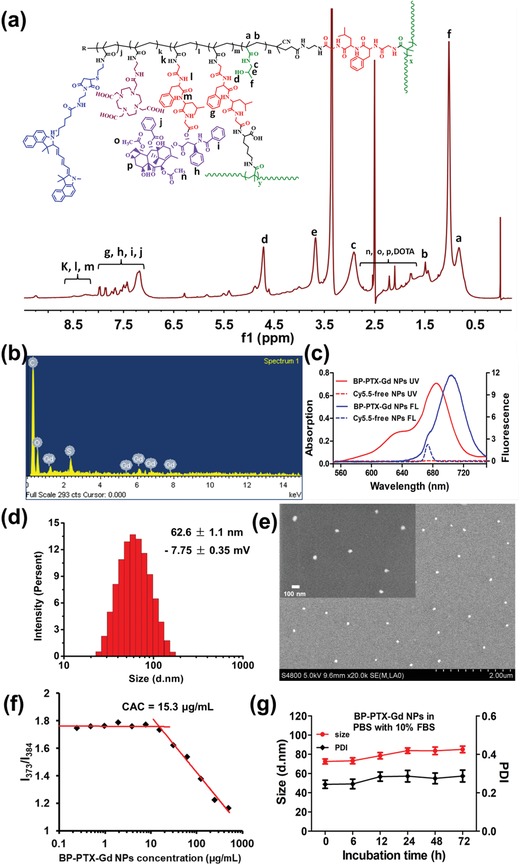
Chemical structure characterization and physicochemical properties of BP‐PTX‐Gd NPs. a) ^1^H NMR of branched pHPMA‐PTX‐DOTA conjugate. Each peak labeled with letters a–p is assigned to the corresponding bonds in the chemical structure of the conjugate. b) EDX spectrum of the branched pHPMA‐PTX‐Gd conjugate. The peak for each element is assigned from the standard references. c) UV–vis and fluorescence spectra of the branched pHPMA‐PTX‐Gd conjugate (dissolved in dimethyl sulfoxide) before and after addition of Cy5.5. d) Size distribution and zeta potential of BP‐PTX‐Gd NPs in aqueous solution determined by DLS. e) SEM image of BP‐PTX‐Gd NPs. f) CAC of BP‐PTX‐Gd NPs. g) Temporal changes in size and PDI of the BP‐PTX‐Gd NPs in PBS with 10% FBS for 72 h. The values are presented as the average ± standard deviation (*n* = 3).

The structure of the as‐prepared BP‐PTX‐DOTA conjugate was characterized by proton nuclear magnetic resonance (^1^H NMR) spectroscopy, energy dispersive X‐ray spectroscopy (EDX), Fourier transform infrared (FTIR) analysis, and amino acid analysis. The molecular weight and polydispersity index (PDI) were determined by size exclusion chromatography. As shown in **Figure** **1**a, the resonance signals at 1.5–0.6 and 5.0–2.8 ppm assigned to the protons of CH—, CH_2_—, CH_3_—, and —OH are associated with pHPMA units. The signals appearing at 2.7–1.6 ppm are ascribed to the protons of CH_2_— in both MA‐DOTA and MA‐GFLG‐PTX units. The aromatic protons derived from MA‐GFLGK‐MA and MA‐GFLG‐PTX units produce peaks at 8.7–7.0 ppm. The elements in the BP‐PTX‐Gd conjugates include N, S, O, and Gd shown in Figure 1b from EDX analysis. Compared to the gadolinium‐free polymer, FTIR spectra showed that the characteristic peak of the carboxyl group (3337.28 and 1717.78 cm^−1^) in the BP‐PTX‐Gd conjugates changed significantly (Figure S2, Supporting Information), indicating that Gd(III) was successfully chelated into DOTA. The characteristic peak of Cy5.5 is observed in both ultraviolet–visible (UV–vis) and fluorescence spectra of Cy5.5‐labeled conjugate compared to the unlabeled conjugate (Figure 1c), confirming that the fluorescent dye has been successfully covalently attached to the conjugate. The amino acid analysis reveals that the molar ratio of glycine (Gly), phenylalanine (Phe), and leucine (Leu) in the conjugate is about 2:1:1 (Table S1, Supporting Information), which is consistent with the amino acid ratio in the monomers, indicating the presence of enzyme‐responsive GFLG tetrapeptides in the polymer backbone. In addition, the molecular weight of the BP‐PTX‐Gd conjugate is 186 kDa with a PDI of 2.30 (Table S1, Supporting Information).

The morphology of the polymer conjugate‐based nanoparticles was detected by dynamic light scattering (DLS) and scanning electron microscope (SEM), respectively. As shown in Figure 1d, the BP‐PTX‐Gd NPs have an intensity‐weighted average hydrodynamic diameter of about 62.6 ± 1.1 nm and a surface charge of −7.75 ± 0.35 mV from DLS measurements, and the size is also confirmed from SEM (about 50 nm, Figure 1e). It is noted that nanoparticles are well dispersed in water from SEM. By varying the nanoparticles concentration (Figure 1f), the BP‐PTX‐Gd conjugate aggregates into nanoparticles at a very low concentration with a critical aggregation concentration (CAC) of ≈15.3 µg mL^−1^. The balance between hydrophilic interaction and hydrophilic interaction is one of the main driving forces for nanoparticle self‐assembly. In this study, the main reason for the formation of nanoparticles by BP‐PTX‐Gd conjugate may be the hydrophilic and hydrophobic effects, because there are many different hydrophilic segments (Gd‐DOTA and HPMA) and hydrophobic segments (GFLG, PTX, Cy5.5) in the polymer structure. In addition, some other driving forces should also be considered, including hydrogen bonding, π–π stacking, dipole interaction, etc., because the hydrophobic part of a branched polymer is composed of multiple domains with different chemical compositions, such as aromatic and aliphatic groups. For stability analysis, the size of BP‐PTX‐Gd NPs slightly increases and the PDI has negligible changes after incubation for 72 h in phosphate buffer saline (PBS) with 10% fetal bovine serum (FBS) (Figure 1g), indicating that the formed nanoparticles may keep its integrated structure in the in vivo circulation system. It is worth noting that either the imaging moiety or PTX is covalently linked to the pHPMA side chain, which further ensures the stability of BP‐PTX‐Gd NPs under physiological conditions. Moreover, the BP‐PTX‐Gd NPs have a nearly neutral surface charge and a size between 10 and 200 nm, which may help achieving low reticuloendothelial cell (RES) uptake, reduced renal excretion, and increased accumulation in tumor sites due to the enhanced permeability and retention (EPR) effect.

### 2.2 Degradation, Drug Release, and Relaxivity of BP‐PTX‐Gd NPs

**Figure 2:**
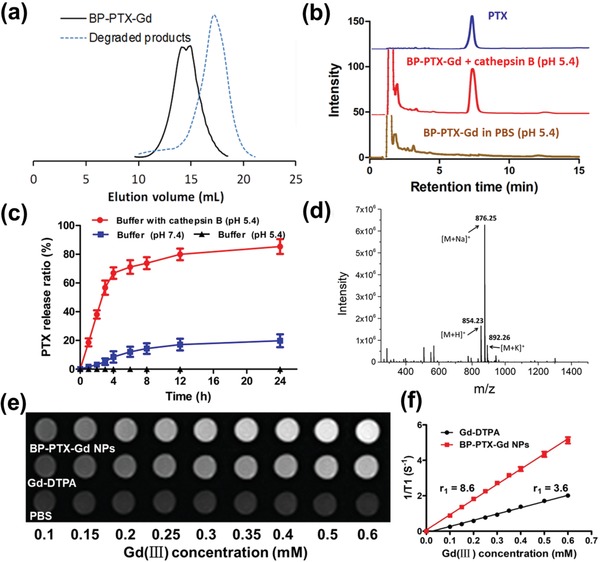
Cathepsin B‐responsive drug release and degradation of BP‐PTX‐Gd NPs. a) SEC profile of BP‐PTX‐Gd conjugates and their degraded products after incubation of the conjugate (3 mg mL^−1^) in PBS (pH 5.4) containing 2.8 × 10^−6^ m cathepsin B for 12 h at 37 °C. b) HPLC chromatograms of BP‐PTX‐Gd NPs with or without cathepsin B (2.8 × 10^−6^ m), free PTX as a control. c) PTX release profiles of the BP‐PTX‐Gd NPs in different buffer solutions at 37 °C. The values are presented as the average ± standard deviation (*n* = 3). d) ESI‐MS analysis of released compounds. e) *T*_1_ weighted MR images of Gd‐DOTA and BP‐PTX‐Gd NPs in PBS with different Gd (III) concentration. f) Water proton longitudinal relaxation rate (1/*T*_1_) of Gd‐DTPA and BP‐PTX‐Gd NPs in PBS as a function of Gd (III) concentration.

The enzyme‐dependent degradation of BP‐PTX‐Gd NPs was investigated by incubation in a simulated tumor cellular microenvironment at a cathepsin B concentration of 2.8 × 10^−6^ m and pH of 5.4. A PBS buffer at pH 7.4 was used as a control. The decrease in the MW of the branched polymers is incubation‐time dependent and the smallest fragments with a MW of around 25 kDa and a smaller PDI are produced after an incubation time of 12 h (Table S2, Supporting Information). A typical peak shift in the SEC chromatogram is shown in **Figure** **2**a at an incubation time of 12 h. After degradation, the peak shifts toward a high value of the elution volume. However, the size and PDI of the conjugate in the control condition are nearly identical after incubation up to 18 h (Table S2, Supporting Information). Previous studies have shown that the HPMA‐based polymer carrier with a high MW can reduce the renal clearance and increase the buildup at the tumor sites, however, the MWs of a polymer carrier above the renal threshold (≈50 kDa) may result in undesirable accumulation of these high MW carriers in the body.[qv: 8c] Biodegradability of these high MW polymer carriers is often sought to address this undesirable accumulation in the issue. In our work, since the crosslinking agent used in the synthesis of the branched pHPMA polymers contains a GFLG tetrapeptide that is cleavable in the presence of cathepsin B, the branched pHPMA polymers are degraded to low MW fragments in the tumor cell microenvironment, which facilitates clearance of the carrier from the body and reduces its potential toxicity.

The accumulated release of PTX from BP‐PTX‐Gd NPs in a simulated tumor cellular microenvironment at 4 h reaches around 67%, much higher than that under a physiological condition (≈8%) (Figure 2c). Compared to the physiological condition, PTX released from the nanoparticles is not detectable in the absence of cathepsin B at pH 5.4. This result is consistent with previous reports: the ester bond between PTX and pHPMA has great stability under a weakly acidic condition.^18^ The released PTX from the nanoparticles has the same peak as the original PTX in both high performance liquid chromatography (HPLC) and electrospray ionization mass spectrometry (ESI‐MS) (Figure 2b,d).

Next, in order to assess the MRI capabilities of the BP‐PTX‐Gd NPs, we analyzed the *T*_1_ weighted spin‐echo MR imaging of BP‐PTX‐Gd NPs and Gd‐DTPA using a clinical 1.5 T MRI scanner. As shown in Figure 2e, with a gradual increase in the Gd(III) concentration, incremental changes in the brightness are clearly observed for both nanoparticles and Gd‐DTPA, which means that the MR signal is positively enhanced with an increased Gd(III) concentration. Compared to Gd‐DTPA, the BP‐PTX‐Gd NPs solution displays significantly enhanced MRI signal contrast at the same Gd(III) concentration. This result is consistent with the widely accepted fact that covalent attachment of Gd(III) complexes to polymers can significantly enhance the MRI contrast.^19^ Quantitative analysis shows that there is a linear relationship of (1/*T*_1_) versus Gd(III) concentration for both BP‐PTX‐Gd NPs and Gd‐DTPA solutions (Figure 2f). The *r*_1_ value of the BP‐PTX‐Gd NPs is 8.6 mm ^−1^ S^−1^, about 2.4 times that of Dd‐DTPA, which further suggests that the BP‐PTX‐Gd NPs significantly enhances the MRI contrast.

### 2.3 Cell and Tumor Spheroids Uptake of BP‐PTX‐Gd NPs

**Figure 3:**
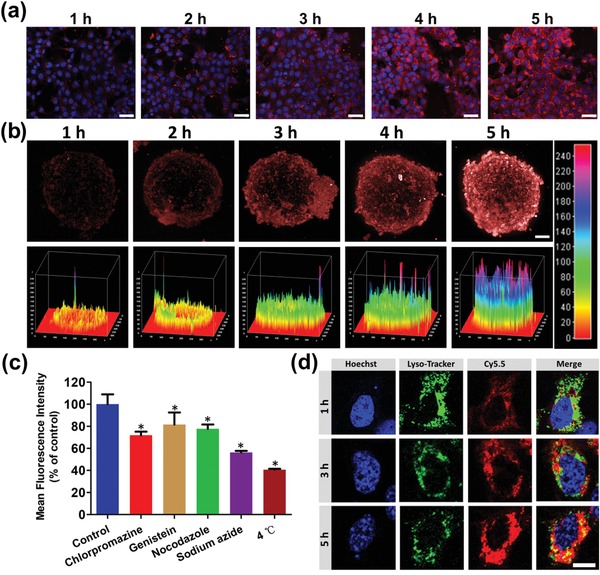
a) CLSM images of 4T1 cells after treatment with Cy5.5‐labeled BP‐PTX‐Gd NPs at different times. Hoechst 33342 (blue) for nuclei, and fluorescence dye Cy5.5 (red) for BP‐PTX‐Gd NPs. The scale is 50 µm. b) CLSM images of 4T1 tumor spheroids after treatment with Cy5.5‐labeled BP‐PTX‐Gd NPs at different times, red represents the fluorescence of Cy5.5. The scale is 100 µm. c) Percentage of BP‐PTX‐Gd NPs uptake by 4T1 cells at different inhibitor conditions (*p < 0.01). d) The lysosomal escape behavior of BP‐PTX‐Gd NPs at different times in 4T1 cells. Hoechst 33342 (blue) for nuclei, lysotracker (green) for lysosomal, and fluorescence dye Cy5.5 (red) for BP‐PTX‐Gd NPs. The scale is 10 µm.

The uptake of BP‐PTX‐Gd NPs by 4T1 cells was studied by confocal laser scanning microscopy (CLSM) and flow cytometry with Cy5.5 as a fluorescent probe. As shown in **Figure** **3**a, as the incubation time of BP‐PTX‐Gd NPs with 4T1 cells is extended from 1 to 5 h, the fluorescence intensity of Cy5.5 (red) in the cytoplasm gradually increases. The results of quantitative analysis by flow cytometry further confirm the time‐dependent internalization of BP‐PTX‐Gd NPs by 4T1 cells (Figure S3, Supporting Information). As shown in Figure 3b, the penetration of BP‐PTX‐Gd NPs into the 4T1 tumor spheroids is also time dependent. As the incubation time increases, the fluorescence intensity in the tumor spheroids increases significantly, which is consistent with the results from quantitative analysis by flow cytometry (Figure S4, Supporting Information). Through tomography, it can be found that BP‐PTX‐Gd NPs are penetrated into the tumor spheroids gradually from the periphery into the core as the incubation time extends (Figure S5, Supporting Information). The fluorescence intensity of BP‐PTX‐Gd NPs in the tumor spheroids has been significantly enhanced after 5 h of incubation compared to 1 h incubation. The above results indicate that the BP‐PTX‐Gd NPs are gradually and efficiently endocytosed by 4T1 cells.

To distinguish the entry pathway of BP‐PTX‐Gd NPs into 4T1 cells, the cellular uptake was re‐examined in the presence of different inhibitors. As shown in Figure 3c, after 4T1 cells were treated at 4 °C or with sodium azide, the uptake rate of BP‐PTX‐Gd NPs decreases to 40.7% and 56.3%, respectively, indicating that the uptake of BP‐PTX‐Gd NPs by cells is an energy‐dependent process. After treatment with nocodazole and genistein, the endocytosed nanoparticles in the 4T1 cells decreases to 77.6% and 81.6%, respectively, indicating that macropinocytosis and caveolin‐mediated endocytosis are involved in the endocytosis process of BP‐PTX‐Gd NPs. It is worth noting that after the 4T1 cells are treated with chlorpromazine, the uptake of BP‐PTX‐Gd NPs is significantly restricted, and the uptake rate drops to 71.9%, indicating that the BP‐PTX‐Gd NPs may be mainly internalized by the clathrin‐mediated endocytosis. The key pathways for cellular uptake of BP‐PTX‐Gd NPs include energy‐dependent endocytosis, clathrin‐mediated endocytosis, caveolin‐mediated endocytosis, and macrocytosis. The multichannel uptake pathways enable BP‐PTX‐Gd NPs to be effectively delivered into 4T1 cells.

Previous studies have shown that nanoparticles internalized by clathrin‐mediated endocytosis will be located in lysosomes.^20^ Therefore, the cellular drug delivery of BP‐PTX‐Gd NPs was monitored by detecting Cy5.5 in the BP‐PTX‐Gd NPs under a CLSM inside the 4T1 cells whose cell nuclei and lysosomes were stained by Hoechst 33342 (blue) and lysotracker (green), respectively. As shown in Figure 3d, after incubation of BP‐PTX‐Gd NPs with 4T1 cells for 1 h, strong red fluorescence is observed in the cytoplasm and it is coincidentally co‐located with the green fluorescence of lysosomes. As the incubation time prolongs, more red fluorescence gradually diffuses into the cytoplasm. At 5 h of incubation, red fluorescence has been significantly enhanced and widely distributed inside the cytoplasm, and it almost completely masks the lysosomal fluorescence. It can be seen that BP‐PTX‐Gd NPs gradually enter the tumor cells through the lysosome endocytic pathway, which may facilitate rapid release of covalently linked PTX under the action of cathepsin B overexpressed in lysosomes, and thus exert its antitumor effect.

### 2.4 Cell Cytotoxicity and Induced Apoptosis

**Figure 4:**
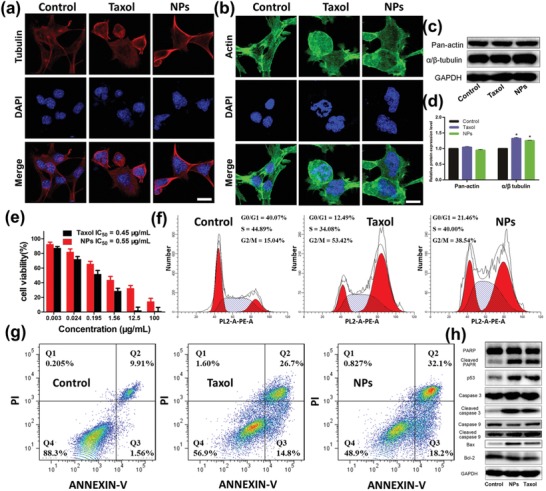
a) Microtubules aggregation and b) microfilament damage of 4T1 cells after treatment with BP‐PTX‐Gd NPs and Taxol for 24 h. Red fluorescence for tubulin antibody, green for actin antibody, and blue for nuclear staining with DAPI. The scale is 10 µm. c) Western blot assay and d) semiquantitative analysis of α/β tubulin and pan‐actin in the 4T1 tumor cells after incubation with Taxol and BP‐PTX‐Gd NPs for 24 h (*p < 0.01 compared to control group). e) Cytotoxicity of BP‐PTX‐Gd NPs and Taxol against 4T1 cells after incubation for 48 h (means ± SD, *n* = 5). f) Cell cycle distribution and g) apoptosis of 4T1 cells after treatment with Taxol and BP‐PTX‐Gd NPs for 24 h. h) Western blot assay of Bcl‐2, Bax, PARP, cleaved PARP, caspase‐3, cleaved caspase‐3, casepase‐9, cleaved caspase‐9, and p53 in the 4T1 tumor cells after incubation with Taxol and BP‐PTX‐Gd NPs for 24 h. Cells treated with PBS were used as a control.

PTX binds to β‐tubulin in the assembled tubulin, maintains the stability of microtubules, and suppresses microtubule dynamics, thereby inhibiting formation of mitotic spindles and leading to cell death. To confirm the effect of PTX on 4T1 cell microtubules, we analyzed the microtubule morphology of cells treated with different formulations under a CLSM. As shown in **Figure** **4**a, in the control group, the microtubules of the cells are evenly distributed in the cytoplasm, while the microtubules of the cells treated with Taxol and BP‐PTX‐Gd NPs display aggregation around the nucleus. The nuclear structure of the control group is intact, however, more than one disintegrated nuclei are observed in the Taxol and BP‐PTX‐Gd NP‐treated group, indicating that mitosis may be inhibited. Furthermore, compared with the filament structure of the control group, the microfilament structure in the drug treatment group appears to be damaged, evidenced with an incomplete microfilament structure, disordered arrangement, points, or a lump shape (Figure 4b). Western blot (WB) analysis reveals that compared with the control group, the amount of α/β‐tubulin in the cells treated by Taxol or BP‐PTX‐Gd NPs increases, while the amount of the pan‐actin protein does not significantly change (Figure 4c,d). Therefore, Taxol or BP‐PTX‐Gd NPs are more likely to destroy the function of microtubules and microfilaments, rather than reduce their contents.

In the lysosomes, BP‐PTX‐Gd NPs are exposed to a tumor cellular microenvironment with overexpressed cathepsin B at an acidic pH. PTX released from the BP‐PTX‐Gd NPs is toxic to 4T1 cells. We used the cell counting kit‐8 (CCK‐8) method to detect the cytotoxicity against 4T1 cells induced by BP‐PTX‐Gd NPs. As shown in Figure 4e, with an increase in the PTX concentration, the cell viability after treatment with Taxol and BP‐PTX‐Gd NPs displays a significant decrease. The IC_50_ value of BP‐PTX‐Gd NPs (0.55 µg mL^−1^) is higher than that of Taxol (0.45 µg mL^−1^), which may be due to a more complex process of entry, trafficking, and drug release of BP‐PTX‐Gd NPs than free PTX.

Furthermore, we used DNA flow cytometry to analyze the effect of PTX released from nanoparticles on cell cycle distribution. As shown in the Figure 4f, the cells in the control group are mainly in the G0/G1 phase and the S phase, and the G2/M phase cells only account for 15.04%. After treatment with Taxol or BP‐PTX‐Gd NPs, the proportion of cells in the G2/M phase significantly increases, increasing to 53.42% and 38.54%, respectively. These results indicate that the PTX released from the nanoparticles has the same efficacy as free paclitaxel, arresting cells in the mitotic G2/M phase of mitosis, resulting in high cytotoxicity. Apoptosis of 4T1 cells incubated with Taxol and BP‐PTX‐Gd NPs by flow cytometry is shown in Figure 4g. Both BP‐PTX‐Gd NPs and Taxol are able to effectively induce 4T1 cell apoptosis compared to the control group without any treatment, and a similar percentage of cells for induced apoptosis and necrosis (51.1% and 43.1%, respectively) is obtained for both groups. To study the mechanism of apoptosis, we used WB to analyze the intracellular content of apoptosis‐related proteins at the level of molecular biology. As shown in Figure 4h, the WB analysis shows that Taxol or BP‐PTX‐Gd NPs treatment downregulates the level of antiapoptotic protein Bcl‐2 in 4T1 cells compared to the control group. At the same time, the level of Bax, cleaved caspase‐3 protein, cleaved caspase‐9, p53 protein, and cleaved PARP protein in the cells is upregulated after treatment with Taxol or BP‐PTX‐Gd NPs, and semiquantitative analysis reveals that a significant difference of the these protein after treatment by both Taxol or BP‐PTX‐Gd NPs compared with the control group (Figure S6, Supporting Information). These results indicate that BP‐PTX‐Gd NPs have a similar mechanism of action as free paclitaxel, which is capable of disturbing cell cycles and activating the corresponding apoptotic pathways, thereby significantly inducing apoptosis.

### 2.5 In Vivo Toxicity and Blood Compatibility of BP‐PTX‐Gd NPs

Excellent biocompatibility is critical to an ideal nanoscale drug delivery system. We first evaluated the hemocompatibility of BP‐PTX‐Gd NPs by detecting the morphology, aggregation, and hemolysis of red blood cells from healthy mice. Red color in the supernatant for red blood cells (RBCs) with BP‐PTX‐Gd NPs at a concentration of up to 4 mg mL^−1^ is not observable (Figure S7a, Supporting Information), and the percentage of hemolysis is around 3.5% at the maximum nanoparticle concentration (4 mg mL^−1^) for 12 h, which is lower than the acceptable percentage of hemolysis (5%) (Figure S7b, Supporting Information). SEM images (Figure S8, Supporting Information) further confirm that there is no obvious aggregation of RBCs after incubation with different concentrations of BP‐PTX‐Gd NPs, and the structure of the erythrocyte membrane is intact in the presence of BP‐PTX‐Gd NPs. As the in vivo blood compatibility influences the nanoparticles biocompatibility and their further application as intravenous agents,^21^ in this study, the blood routine analysis and blood biochemical studies showed the BP‐PTX‐Gd NPs have good blood compatibility (Figures S9 and S10, Supporting Information).

Furthermore, to evaluate the potential toxicity of BP‐PTX‐Gd NPs to normal tissues and organs, healthy mice were treated with BP‐PTX‐Gd NPs for 21 d. The treated mice do not exhibit abnormal symptoms and behaviors (such as weight loss and listlessness, etc.) during the entire experiment period. The hematoxylin‐eosin (H&E) staining of the main organs of the mice in the BP‐PTX‐Gd NPs treatment group does not reveal significant pathological changes in each organ (Figure S11, Supporting Information). Inherent toxicity and off‐target accumulation of the theranostic nanoparticles are major concerns for clinical applications. We have demonstrated low in vivo toxicity of BP‐PTX‐Gd NPs to major organs and great hemocompatibility. Excellent biosafety of the nanomedicine lays the solid foundation for its multimodal functions. The biosafety may stem from its unique structure and degradation in the tumor specific microenvironment as pHPMA polymers have been intensively studies as a biocompatible polymer carrier.^22^

### 2.6 Pharmacokinetics and Biodistribution

**Figure 5:**
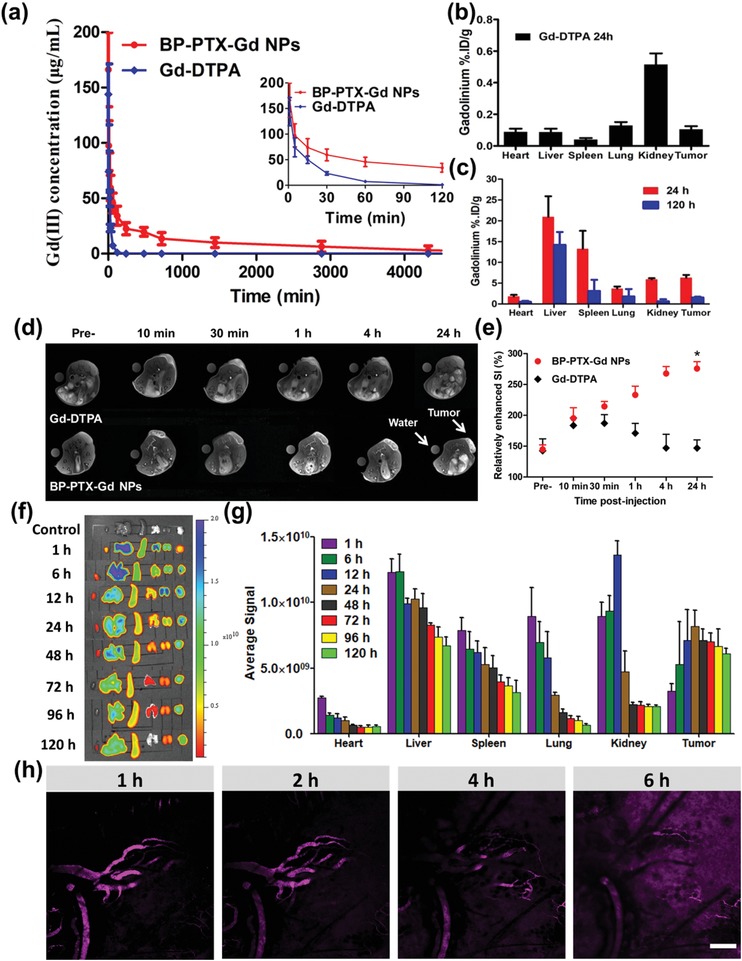
a) In vivo pharmacokinetic data of Gd (III) concentration after intravenous injection of BP‐PTX‐Gd NPs and Gd‐DTPA, respectively (means ± SD, *n* = 5). b) Biodistribution of Gd(III) in major organs and tumors at 24 h postinjection of Gd‐DTPA in 4T1 tumor‐bearing mice (*n* = 5). c) Biodistribution of Gd(III) in major organs and tumors at 24 and 120 h postinjection of BP‐PTX‐Gd NPs in 4T1 tumor‐bearing mice (means ± SD, *n* = 5). d) In vivo MR images of mice bearing subcutaneous 4T1 tumors after intravenous injection of BP‐PTX‐Gd NPs and Gd‐DTPA at different time points. e) Relative MR enhanced signal intensity (SI) in tumor sites after injection of BP‐PTX‐Gd NPs and Gd‐DTPA. *p < 0.01 compared to Gd‐DTPA (means ± SD, *n* = 5). f) Ex vivo fluorescent imaging of tumors and major organs harvested from the Cy5.5‐labled BP‐PTX‐Gd NP‐treated mice. g) Average signals from major organs and tumors at different time points after injection of Cy5.5‐labled BP‐PTX‐Gd NPs (means ± SD, *n* = 5). h) Confocal microscopy of time‐dependent extravasation and penetration of BP‐PTX‐Gd NPs (purple) in 4T1 tumors at intervals after the injection. Scale bars = 200 µm.

**Figure** **5**a shows the plasma pharmacokinetic profiles of Gd(III) in BP‐PTX‐Gd NPs and Gd‐DTPA‐treated mice. For the small molecule Gd‐DTPA, the concentration of Gd(III) in the blood descends rapidly to a very low level within 1 h. In contrast, the concentration of Gd(III) in the blood of mice treated with BP‐PTX‐Gd NPs reduces relatively slower. The in vivo blood circulation time of BP‐PTX‐Gd NPs is remarkably prolonged, and the half‐life time (*t*_1/2_) for BP‐PTX‐Gd NPs is 50.5‐fold longer than that of Gd‐DTPA (13.63 and 0.27 h, respectively) (Table S3, Supporting Information). In addition, the results of angiography in mice obtained by MRI are consistent with those of pharmacokinetic experiments. Compared with small molecule contrast agents Gd‐DTPA, BP‐PTX‐Gd NPs are still visible in the blood circulation system up to 4 h after injection, and blood vessel structure is also clearly distinguished in the BP‐PTX‐Gd NP‐injected group (Figure S12, Supporting Information). The prolonged circulation time contributes to an increase in passive accumulation of the nanomedicine at the tumor site, resulting in enhanced imaging and therapeutic effects. The significantly prolonged blood half‐life (*t*_1/2_) of BP‐PTX‐Gd NPs is due to a high MW of the nanomedicine, stable linkers in the chemical structure, a slightly negatively charged surface, and a branched structure with excellent chain flexibility and deformability.

The Gd(III) content in the heart, liver, spleen, lung, kidney, and tumor of tumor‐bearing mice at 24 h postadministration of Gd‐DTPA is shown in Figure 5b. The highest Gd(III) content is found in the kidney after 24 h, and it is only 0.5%. In contrast, a higher level of Gd(III) content is detected in liver and spleen in the BP‐PTX‐Gd NPs group (20.9% and 13.2%, respectively) (Figure 5c), which is consistent with previous reports that pHPMA polymers with a high MW are prone to accumulation in the liver and spleen.^23^ Furthermore, the tumor in the BP‐PTX‐Gd NP‐treated mice contains a relatively high Gd(III) content of 6.3%, which supports that the theranostic nanomedicine is effectively accumulated at tumor sites. To further evaluate the metabolism of BP‐PTX‐Gd NPs in mice, the Gd(III) content at each organ and tumor in mice was measured at 120 h postadministration. As shown in Figure 5c, the Gd(III) level in all tissues has significantly decreased in comparison with that on the first day postinjection. The low Gd(III) residues in mice indicate that BP‐PTX‐Gd NPs may be excreted from body, which is consistent with the results of ex vivo fluorescence imaging, and this clearance may be attributed to biodegradability of the nanomedicine. This is also of great significance for confirming the biosafety of the nanoscale branched pHPMA‐based MRI contrast agent.

### 2.7 MR and Fluorescence Imaging

The imaging function of BP‐PTX‐Gd NPs was further evaluated at tumor sites in BALB/c female mice bearing subcutaneous 4T1 tumors by a 3 T scanner. As shown in Figure 5d, after 30 min of administration, the tumor signal intensity (SI) in both BP‐PTX‐Gd NPs and Gd‐DTPA‐treated groups remarkably increases. The relatively enhanced MRI SI of BP‐PTX‐Gd NPs and Gd‐DTPA is 214% and 191%, respectively, in comparison with an SI of around 150% at preinjection (Figure 5e). However, for the Gd‐DTPA group, the SI at the tumor site dramatically decreases when the administration time exceeds 30 min. At 4 h postadministration, SI has reduced to the level before administration. In contrast, for mice injected with BP‐PTX‐Gd NPs, the SI at the tumor site gradually increases as time increases during the experiment, and the highest relatively enhanced MRI value (about 275%) is reached after 24 h of administration. Compared to the small molecule Gd‐DTPA, which is rapidly eliminated from the body via renal excretion, BP‐PTX‐Gd NPs have a bright contrast intensity, and the MRI signal maintains its brightness after 24 h postinjection. This allows detection of the injected nanomedicine at the tumor sites in a noninvasive manner.

Cy5.5 is a fluorescence probe, which has a deeper tissue penetration and a lower autofluorescence signal,^24^ and Cy5.5‐labeled nanoparticles are tracked by detection of Cy5.5 fluorescence for their tempospatial distribution in a specific organ or tissue, which allows a better understanding of the dynamic distribution of nanomedicine in the organs and tissues, and possible excretion pathways.^25^ In this work, ex vivo fluorescence images and quantitative fluorescent signals of main organs and tumors of mice at different time points after injection of Cy5.5‐labeled BP‐PTX‐Gd NPs are shown in Figure 5f,g, respectively. As the time prolongs after administration, the fluorescence intensity at the tumor site increases to a peak at 24 h postinjection and then it starts to gradually decay, while the fluorescence intensity in the liver, spleen, lung, and heart has a monotonously decreasing trend. Interestingly, the fluorescence intensity at the kidney reaches the highest level at 12 h postinjection. The imaging results imply that BP‐PTX‐Gd NPs may gradually accumulate at the tumor site through the EPR effect and the degraded NPs may be excreted by kidney metabolism.

Further, we examined the vascular permeability of BP‐PTX‐Gd NPs. As shown in Figure 5h, a very pronounced fluorescent signal of Cy5.5 is observed in the blood vessels of the tumor site at 1 h after administration through the tail vein, and the signal is mainly located in the tumor blood vessels. With prolongation of the postinjection time, the boundary between the vascular region and the nonvascular region becomes blurred. A gradual increase in the fluorescence intensity in the nonvascular region may be associated with leakage of the nanoparticles from the blood vessel to the tumor tissue through the EPR effect and accumulation of these nanoparticles in the tumor site. After 6 h of administration, the fluorescence intensity in the tumor area is markedly strengthened, and it is almost impossible to distinguish between the vascular and nonvascular regions. This result indicates that BP‐PTX‐Gd NPs can rapidly leak from the blood vessel to the tumor tissue through abnormal tumor vascular structures and accumulate in the tumor.

### 2.8 Imaging‐Guided Antitumor Therapy

**Figure 6:**
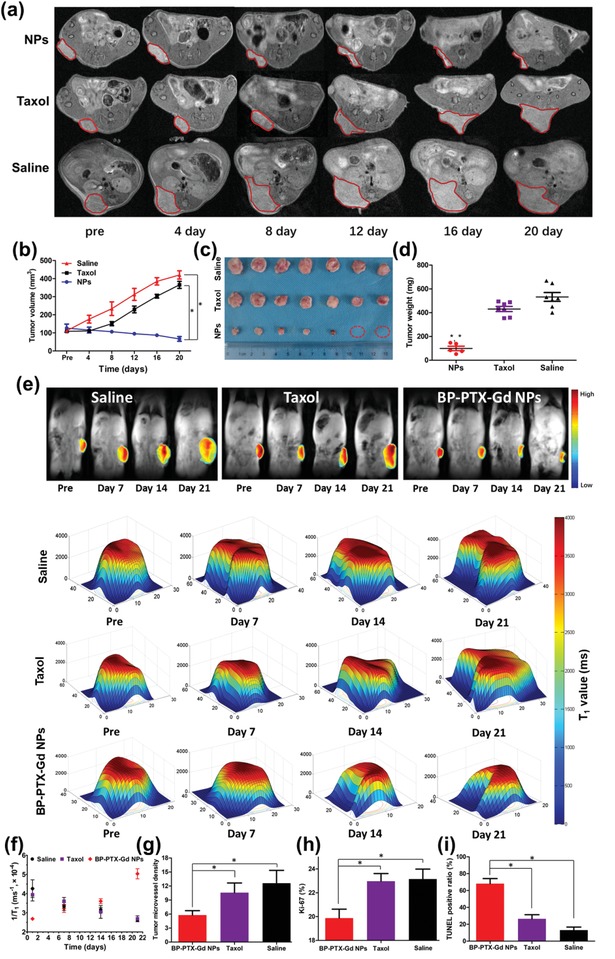
a) Representative MR images of tumor size at different time points for each treatment group of mice (tumors marked by red lines). b) Quantitative tumor volume changes of mice obtained by MRI after being treated with saline, Taxol, and BP‐PTX‐Gd NPs (*n* = 7, *p < 0.001 vs saline, *p < 0.001 vs Taxol). c) Images of the tumors harvested from the mice 21 d after the treatment. d) The tumor weight of tumor‐bearing mice after treatment (*p < 0.001 vs saline, *p < 0.001 vs Taxol). e) Distribution of *T*_1_ value of tumor at different time points after injection. The results are a representative of three separate experiments. *T*_1_‐map sequence: SE, TR = 15–500 ms, TE = 2.0 ms, matrix = 256 × 256, slice thickness = 2.0 mm, gap = 0.0 mm, and field of vision = 160 × 160 mm. f) *T*_1_‐mapped images of mouse carcinoma in situ at different time points after injection with different formulations. Immunohistochemical analysis of g) CD31, h) Ki‐67, and i) TUNEL assays for 4T1 tumors (mean ± SD, *p < 0.01).

In vivo therapeutic efficacy was further determined in 4T1 tumor‐bearing mice treated with BP‐PTX‐Gd NPs or Taxol, while saline was used as a control. The change in the tumor volume was accurately detected by a high resolution 7.0 T small animal MRI scanner during the treatment. As shown in **Figure** **6**a, after three administrations every 7 d, the MRI snapshots reveal that the tumor volume in the Taxol‐treated group and the saline group gradually becomes larger during the treatment. In contrast, the tumor volume in the BP‐PTX‐Gd NP‐treated group significantly shrinks. These results are consistent with the relative tumor volume changes measured by the vernier caliper (Figure S13a, Supporting Information). The tumor volume is also shown quantitatively in Figure 6b. Taxol displays a weak inhibitory effect on tumor growth, and the average tumor volume in mice gradually increases from 108 to 360 mm^3^ throughout the treatment period. In contrast, BP‐PTX‐Gd NPs significantly inhibit the growth of tumors. The average tumor volume in the NP‐treated group before administration is about 127 mm^3^, and the tumor volume starts to gradually shrink after administration. On the 20th day, the average tumor volume reduces to 66 mm^3^, and the tumors in 2 mice are completely eradicated (Figure 6c), which may be attributed to passive accumulation of the branched pHPMA polymer‐carried PTX at the tumor sites to increase the drug dose, and rapid release of PTX from the polymers in the lysosomes to exert its therapeutic effects after uptake through the endocytosis pathways. MRI is able to accurately monitor changes in the mouse tumor volume during treatment. This detection method has obvious advantages compared with measurement of tumor size by vernier caliper: it can measure the volume changes of irregular shaped tumors more accurately, and provide real‐time feedback for the treatment of tumors. Moreover, no significant body weight loss is observed for mice treated with BP‐PTX‐Gd NPs (Figure S13b, Supporting Information), which further indicates negligible systemic toxicities of BP‐PTX‐Gd NPs.

After the experiment, all mice were euthanized to harvest the major organs and tumors. The tumors were weighed (Figure 6d), and the tumor growth inhibition (TGI, %) calculated from the tumor weight is presented in Figure S13c in the Supporting Information. The TGI up to 81.3% is obtained from BP‐PTX‐Gd NP‐treated mice, while only 19.0% for Taxol‐treated mice. To further evaluate the antitumor effects of BP‐PTX‐Gd NPs, we changed the method of administration to once every 4 d (4 times in total). After increasing the therapeutic dose, the therapeutic effect of the nanoparticles is further improved, and 4 out of 7 tumors have been completely eliminated. The TGI reaches 93%, and no obvious systemic toxicity is observed (Figure S14, Supporting Information). Compared to our previously reported branched polymeric prodrug‐based nanoparticles (TGI 50–85%),^23^, ^25^ this system can significantly inhibit the tumor growth and some tumors can be completely eradicated, resulting in significantly enhanced therapeutic indexes. This may be due to optimized molecular structure, superior tumor targeting, and rapid drug release with enzyme responsiveness.

Previous studies have shown that the *T*_1_ values of the tumor site is related to its internal environment, so the therapeutic effect was also examined by monitoring changes in the *T*_1_ values of the tumor site.^26^ Figure 6e shows pseudocolor maps of tumor *T*_1_ values during treatment in different experimental groups. Figure 6f summarizes the changes in *T*_1_ values of tumor sites in each group of mice treated by different formulations through a clinical 3.0 T MRI scanner. During the entire treatment period, the *T*_1_ values detected in the BP‐PTX‐Gd NP‐treated group reveals a gradual increase as the treatment time extends, while the *T*_1_ values of the tumor site of the saline group and the Taxol group displays the opposite trend. Because the tumor volume in the BP‐PTX‐Gd NP‐treated group gradually decreases during the treatment process, supply of water and blood to the tumor extracellular matrix is reduced. The change in the tumor environment leads to an increase in the *T*_1_ value of the tumor site. In contrast, the tumor volume of the Taxol group and the saline group gradually expands during the treatment, and the extracellular water and blood supply to the tumor continuously increases, resulting in a continuous decrease in the *T*_1_ value.^27^ These results are consistent with the trend of tumor volume changes monitored by MRI, further demonstrating that BP‐PTX‐Gd NPs can achieve effective tumor therapy in vivo.

Subsequently, we investigated the inhibition mechanism of BP‐PTX‐Gd NPs by analyzing angiogenesis (CD‐31, an endothelium cell marker), cell proliferation (Ki‐67), and apoptosis (TUNEL) in tumor tissues by immunohistochemistry (IHC) staining (Figure S15, Supporting Information). The tumor microvessel density (MVD) derived from imaging analysis of the CD‐31 stained tissue after injection of BP‐PTX‐Gd NPs is 5.5, which is remarkably lower than that in the Taxol‐treated group and the control group (10 and 11, respectively, *p* < 0.01) (Figure 6g). Ki‐67 staining reveals that the tumor cell proliferation rate treated by BP‐PTX‐Gd NPs is significantly lower than that of both Taxol and control groups (Figure 6h). After TUNEL staining (Figure 6i), treatment with BP‐PTX‐Gd NPs efficiently increases the percentage of TUNEL‐positive tumor cells (apoptotic cells). These above results demonstrate that BP‐PTX‐Gd NPs effectively inhibit tumor angiogenesis and tumor cell proliferation, and induce tumor cell apoptosis, thus they have an excellent antitumor efficacy. Furthermore, histological analysis was used to detect cancer metastasis and potential toxicity of the theranostic nanomedicine. As shown in Figure S16 in the Supporting Information, tumor metastasis is observed in the lung of the mice in the saline group and the Taxol‐treated group. In contrast, no tumor metastasis is detected in the BP‐PTX‐Gd NP‐treated group. No obvious pathological changes are observed in major organs after treatment by BP‐PTX‐Gd NPs, Taxol, and saline at day 21, indicating excellent biosafety of BP‐PTX‐Gd NPs.

## 3 Conclusion

In summary, biodegradable branched pHPMA‐PTX‐Gd‐Cy5.5 conjugate synthesized via “one‐pot” RAFT polymerization is constituted of branched pHPMA as a carrier, an anticancer drug, and two imaging agents: Gd‐DOTA as a contrast agent for MR imaging and Cy5.5 as a near infrared fluorescent dye for fluorescence imaging. Cathepsin B‐responsive tetrapeptide GFLG for bridging pHPMA and PTX‐pHPMA polymer chains is cleaved in a tumor microenvironment, leading to generation of small MW polymeric segments and PTX release specifically in the tumor cells with an equivalent potency to free PTX. BP‐PTX‐Gd NPs have an extended in vivo circulation time and are effectively accumulated at the tumor site, evidenced by pharmacokinetic analysis, MRI, and fluorescence imaging. BP‐PTX‐Gd NPs significantly increase the relaxation efficiency and enhance contrast intensity in comparison with small MW contrast agents. BP‐PTX‐Gd NPs have performed much better than Taxol in eliminating 4T1 murine breast cancer in the mice while they are safe to the healthy tissue/organs. These results demonstrate a promising potential of the as‐synthesized nanoparticles as a theranostic polymer‐based nanoscale medicine for treating a variety of cancer diseases.

## 4 Experimental Section

Materials, methods, experiments, other data, and associated references are available in the Supporting Information. All animal operation procedures were carried out with the approval of the ethics committee of West China Hospital, Sichuan University (No. 2018148A and 2018150A).

## Conflict of Interest

The authors declare no conflict of interest.

## Supporting information

Supporting Information
