# Supplementary material for: A Nanostrategy for Efficient Imaging‐Guided Antitumor Therapy through a Stimuli‐Responsive Branched Polymeric Prodrug
Source: Adv Sci (Weinh). 2020 Jan 31;7(6):1903243. doi: 10.1002/advs.201903243 (PMC7080516; doi:10.1002/advs.201903243)
Supplement: Supplementary file 1 — Supporting Information [file ADVS-7-1903243-s001.pdf]

Copyright WILEY-VCH Verlag GmbH & Co. KGaA, 69469 Weinheim, Germany, 2020.

ADVANCED SCIENCE
Open Access

Supporting Information

for *Adv. Sci.,* DOI: 10.1002/advs.201903243

A Nanostrategy for Efficient Imaging-Guided Antitumor Therapy through a Stimuli-Responsive Branched Polymeric Prodrug

*Hao Cai, Xinghang Dai, Xiaoming Wang, Ping Tan, Lei Gu, Qiang Luo, Xiuli Zheng, Zhiqian Li, Hongyan Zhu, Hu Zhang, Zhongwei Gu, Qiyong Gong, and Kui Luo**

Copyright WILEY-VCH Verlag GmbH & Co. KGaA, 69469 Weinheim, Germany, 2020.

Supporting Information

A nanostrategy for efficient imaging-guided anti-tumor therapy through a stimuli-responsive branched polymeric prodrug

*Hao Cai, Xinghang Dai, Xiaoming Wang, Ping Tan, Lei Gu, Qiang Luo, Xiuli Zheng, Zhiqian Li, Hongyan Zhu, Hu Zhang, Zhongwei Gu, Qiyong Gong, Kui Luo**

Dr. H. Cai, Mr. X. M. Wang, Dr. P. Tan, Ms. L. Gu, Mr. Q. Luo, Dr. X. L. Zheng, Mr. Z. Q. Li, Prof. H.Y. Zhu, Prof. Z. W. Gu, Prof. Q. Y. Gong, Prof. K. Luo
Huaxi MR Research Center (HMRRC), Department of Radiology, Functional and molecular imaging Key Laboratory of Sichuan Province, West China Hospital, Sichuan University, Chengdu 610041, China
*E-mail: luokui@scu.edu.cn

Prof. Z. W. Gu, Prof. K. Luo
National Engineering Research Center for Biomaterials, Sichuan University, Chengdu 610064, China

Prof. H. Zhang
Amgen Bioprocessing Centre, Keck Graduate Institute, CA 91711, USA

Mr. X. H. Dai
West China School of Medicine, Sichuan University, Chengdu 610041, China

1. EXPERIMENTAL SECTION

1.1. Materials and methods

4-Dimethylaminopyridine (DMAP), N,N′-Dicyclohexylcarbodiimide (DCC), N,N-diisopropylethylamine (DIEA), 1-hydroxybenzotriazole (HOBt) and N,N,N′,N′-tetramethyl-(1H-benzotriazol-1-yl)uronium hexafluorophosphate (HBTU) were bought from GL Biochem (Shanghai, China). Gadolinium chloride hexahydrate ($GdCl_3 \cdot 6H_2O$), dithiothreitol (DTT) and cathepsin B were procured from Sigma-Aldrich (St. Louis., MO, USA). Taxol® was purchased from Baoman Biotechnology (Shanghai). Cyanine5.5 maleimide was bought from Lumiprobe (Hallandale Beach, FL). All other reagents were commercially available and used as received, unless noted otherwise. Monomers *N*-(2-hydroxypropyl) methacrylamide (HPMA),[1] *N*-[2-(2-pyridyldithio)]ethyl methacrylamide (PTEMA),[2] MA-DOTA,[3] MA-GFLG-PTX,[4] MA-GFLG-CTA[5] and MA-GFLGK-MA[5] were prepared and characterized as previously described.

Proton nuclear magnetic resonance (^{1}H NMR) was carried out on a 400 MHz Bruker Advanced Spectrometer. The structures of various monomers were characterized by electrospray ionization mass spectrometry (ESI-MS, TSQ Quantum Ultra, Themo Scientific, USA). The weight- and number-averaged molecular weights and polydispersity (*Mw/Mn*) were obtained by size-exclusion fractionation on a Superose 6 HR10/30 column using an ÄKTA fast protein liquid chromatography (FPLC) system (GE Healthcare). Fourier transform infrared (FTIR) spectroscopy was used to confirmed the structures of polymer precursors and final products. Energy dispersive X-Ray spectroscopy (EDX) was used to determine the elements contained in the conjugate. Paclitaxel (PTX) content of conjugate was determined by high performance liquid chromatography (HPLC) using water/acetonitrile (1:1, v/v) at 1.0

mL/min and detected by UV absorbance at 227 nm. The cyanine5.5 (Cy5.5) labelled in conjugate was measured by a fluorescence spectrophotometer (Hitachi F-7000, Japan) after dissolved in dimethyl sulfoxide (DMSO) (λ_{ex} = 676 nm, λ_{em} = 708 nm). The Gd(III) content in the final product was determined by inductively coupled plasma mass spectrometry (ICP-MS) measurements.

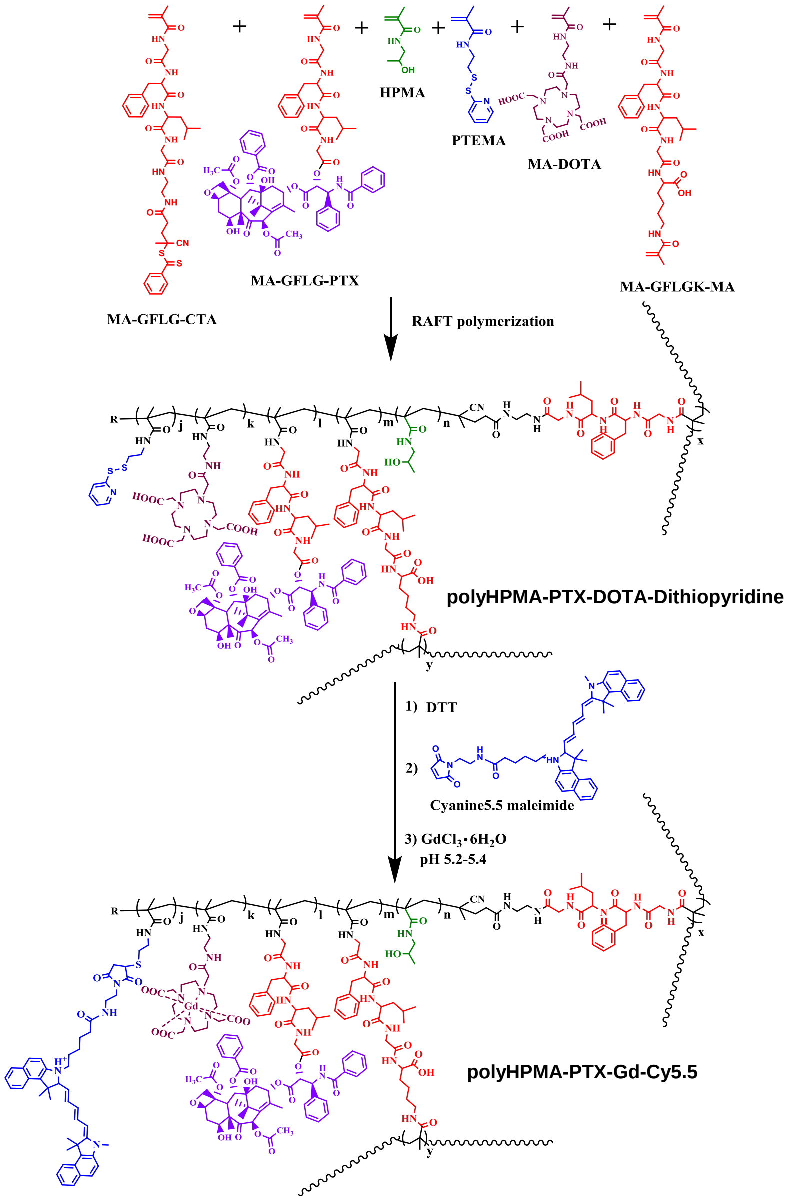

Figure S1. Synthesis procedure of the branched pHPMA-PTX-Gd conjugate. Branched pHPMA-PTX-DOTA was synthesized through one-step RAFT polymerization, allowing attachment of PTX through the enzyme-responsive linker, covalently binding of Cy5.5 by thiol-ene click reaction, and chelation with DOTA on the side chain of branched pHPMA polymers.

1.2. Fabrication of branched polyHPMA-PTX-Gd-Cy5.5 conjugates

Under argon atmosphere, in a 25 mL reaction flask equipped with a magnetic stirring bar, HPMA (805.5 mg, 5.63 mmol), MA-DOTA(1.16 g, 2.25 mmol), MA-GFLG-PTX(583 mg, 0.45 mmol), PTEMA (38 mg, 0.15 mmol), MA-GFLGK-MA(98.4 mg, 0.15 mmol) and MA-GFLG-CTA(64.1 mg, 67 μmol) were dissolved in 12 mL of mixed solution (H_2O : CH_3OH = 1 : 1, v/v) containing VA044 (8.1 mg, 25 μmol). Then, the reaction flask was placed into an ice bath and purged for 30 min to remove oxygen. Under stirring, the reaction flask was moved to an oil bath with a temperature of 45 °C. After reacting for 16 h in the dark, the polymerization was stopped by quenching with liquid nitrogen. Subsequently, the solution was slowly added dropwise to an ether/acetone (1: 1) solution under vigorous stirring, and the precipitate was collected. The precipitate was dissolved with a small amount of methanol and precipitated again with acetone, the precipitate was collected and dried to give a pale pink solid. The polymer samples were further fractionated/purified by size-exclusion fractionation using an ÄKTA fast protein liquid chromatography (FPLC) system (GE Healthcare). The column (Superose 6 HR10/30) were eluted using sodium acetate buffer containing 30% acetonitrile (v/v, pH 6.5). The separated product was subjected to dialysis and lyophilization to obtain the product (polyHPMA-PTX-DOTA-Dithiopyridine) conjugate (1.24 g), a yield of 45%.

To remove the dithiopyridine group, branched polyHPMA-PTX-DOTA-Dithiopyridine conjugates (1.0 g) was dissolved in 5 mL distilled water, under stirring, 200 mg dithiothreitol (DTT) was added. The mixed solution continued to react for 6 h and then dialyzed with distilled water (MWCO = 2 kDa). After lyophilization, the product obtained from the previous step was dissolved in 10 mL DMSO. 1 mL of

DMSO containing 10 mg Cyanine5.5 maleimide was slowly added to the solution under stirring. After 12 h stirring in the dark, the solution was dialyzed against distilled water and lyophilized to obtain branched polyHPMA-PTX-DOTA-Cy5.5 conjugates (slightly blue power, 988 mg).

In order to chelate Gd (III), branched polyHPMA-PTX-DOTA-Cy5.5 conjugate (800 mg) and $GdCl_3 \cdot 6H_2O$ (200 mg) were dissolved in deionized water (20 mL), and then the pH of the solution was adjusted to 5.2~5.4 with NaOH (0.1M) under stirring. After stirring in the dark for 24 h, the mixture was dialyzed for 15 h to completely remove excess Gd(III). Finally, the resulting solution was lyophilized to give the final product, branched polyHPMA-PTX-Gd-Cy5.5 conjugates (slightly blue solid, 810 mg). The contents of PTX, Cy5.5 and Gd(III) in the final conjugates were 7.6wt%, 0.7 wt% and 6.5 wt%, respectively.

1.3. Preparation of BP-PTX-Gd NPs and determination of critical aggregation concentration (CAC)

30 mg of BP-PTX-Gd conjugates dissolved in DMSO (2 mL) was slowly added dropwise to distilled water (10 mL) with stirring. Subsequently, DMSO was removed by dialysis (MWCO 2 kDa) with distilled water for 24 h at 4 °C. After lyophilization, the BP-PTX-Gd nanoparticles (BP-PTX-Gd NPs) were obtained. Next, pyrene was used as a probe to detect the CAC of BP-PTX-Gd. Briefly, pyrene dissolved in acetone (10 μg/mL, 25 μL) was placed in a 10 mL vial. Subsequently, 2 mL of BP-PTX-Gd solution at different concentrations (range of 0.0005~1 mg/mL) was added to each vial after acetone was evaporated. The solutions were then equilibrated at room temperature and kept in the dark for 12 h. The CAC value of BP-PTX-Gd copolymer was determined by measuring the change in the fluorescence intensity (λ_{ex} = 330 nm).

1.4. Size, zeta potential and morphology

The size distribution and zeta potential of BP-PTX-Gd NPs in the dispersion state were determined by dynamic light scattering (DLS). The BP-PTX-Gd concentrations in distilled water were kept at 3 mg/mL and measurements were performed at room temperature. The stability of BP-PTX-Gd NPs in phosphate buffer saline (PBS) with 10% fetal bovine serum (FBS) was determined according to the same conditions as above. The morphology of nanoparticles formed by BP-PTX-Gd was determined by scanning electron microscope (SEM). The BP-PTX-Gd was re-dissolved in distilled water (1 mg/mL) and then coated on silicon wafers and air-dried. The dried samples were finally observed under a microscope.

1.5. Degradation and drug release of BP-PTX-Gd NPs

Degradation of BP-PTX-Gd NPs under a simulated tumor cell microenvironment (McIlvaine's buffer (50 mM citrate/0.1 M phosphate, 2 mM ethylene diamine tetraacetic acid (EDTA), pH 5.4)) with cathepsin B (2.8 μM) was examined at a BP-PTX-Gd concentration of 5 mg/mL for 18 h at 37 °C. The molecular weight changes at different time intervals were monitored by SEC measurements. The McIlvaine's buffer in absence of cathepsin B and a PBS buffer at pH 7.4 were used as controls.

The release of PTX from BP-PTX-Gd NPs were monitored in three media: McIlvaine's buffer (pH 5.4) with 2.8 μM cathepsin B, McIlvaine's buffer (pH 5.4) without cathepsin B, and McIlvaine's buffer (pH 7.4) without cathepsin B. Samples of BP-PTX-Gd NPs (5 mg/mL) were incubated in different buffers for 24 h at 37 °C in a shaker. At predetermined time intervals, 100 μL of sample solution was removed and diluted with an equal volume of methanol. The absorbance of the solution at 227 nm by HPLC was read and the cumulative release percentage (%) was calculated. The

released component was also identified by ESI-MS. The release experiments were carried out in triplicate and the data represent as mean ±SD.

1.6. T_1 Relaxivity of DTPA-Gd and BP-PTX-Gd NPs

The conjugate was dissolved in 0.1 M PBS aqueous solution at different Gd(III) concentrations ranging from 0.10 to 0.60 mM, and the *in vitro* relaxivity of the HPMA-Gd-PTX conjugate was determined from a clinical 1.5 T magnetic resonance imaging (MRI) scanner (Siemens Sonata, Germany) at room temperature, while Gd-DTPA was used as a control. For the T_1-weighted images of the conjugates, the following parameters were employed: the spin-echo method, TE = 8.7 ms; TR = 25, 30, 50, 70, 90, 110, 150, 170, 190, 210, 250, 300, 400, 600, 700, and 800 ms; Fov = 200 mm; slice thickness = 2.0 mm; matrix dimensions = 256 × 256.[3] The r_1 value was determined from the slope in the plot of 1/relaxation time (s^{-1}) *versus* the Gd(III) concentration.

1.7. Tumor cells culture and animal model

The 4T1 murine breast cancer cell line were acquired from Shanghai Institutes for Biological Sciences (China) and cultured in complete RPMI 1640 medium at 37 °C in 5% CO_2. Female BALB/c mice (5-8 weeks old, 20 ± 2 g) were purchased from Chengdu DaShuo Biological Technology Co., Ltd (China) and used for the tumor model. All animal operation procedures were carried out with the approval of the ethics committee of West China Hospital, Sichuan University (No. 2018148A and 2018150A). A 4T1 breast cancer xenograft mouse model was established by injecting 5×10^5 4T1 cancer cells subcutaneously at the right lower back of female BALB/c mice. The tumor volume (mm^3) was calculated as ½ × L × W^2, where L and W refer to the lengh and width of the tumor, respectively.

1.8. Cytotoxicity, cell cycle, apoptosis and western blot assays

For cytotoxicity assay, 4T1 cells were seeded in 96-well plates at a density of 5 × 10^3 cells per well and incubated for 24 h. After removing culture medium, Taxol® and BP-PTX-Gd NPs in complete RPMI medium with different PTX concentrations (0.003~100 μg/mL) were added. After 48 h incubation, the cells were incubated with 100 μL of culture medium containing 10% CCK-8 (v/v) for another 2 h, the absorbance at 450 nm was recored by a Varioscan Flash microplate reader (Themo Fisher Scientific, USA). Graphpad Prism 5 software was selected to calculate the IC_{50} values for each group. Cells without treatment were used as a control.

For cell cycle study, 4T1 cells were seeded in a 6-well plate at a density of 1.5 × 10^5 cells per well. After incubating for 24 h, cells were treated with Taxol® and BP-PTX-Gd NPs at a PTX concentration equal to their IC_{50} values for further 24 h at 37 °C. Subsequently, cells in each group were harvested, fixed with 70% ethanol at -4 °C overnight. The treated cells were washed by PBS, incubated with RNase A, followed by PI (0.1 mg/mL) staining. Cell cycle distribution was analyzed using flow cytometry, and the data was processed by ModFit software.

For apootosis study, 4T1 cells in the logarithmic growth phase were seeded in a 6-well plate at a density of 1.5 × 10^5 cells per well. After incubating for 24 h, cells were treated with Taxol® and BP-PTX-Gd NPs at a PTX concentration equal to their IC_{50} values for further 24 h at 37 °C. All cells were harvested, suspended in a binding buffer, stained with fluorescein isothiocyanate (FITC)-labeled annexin V (annexin V-FITC) and propidium iodide (PI) for 20 min in the dark, and analyzed by flow cytometry.

For western blot assay, the cells were treated as described above and subsequently washed with PBS, and then lysed in RIPA Lysis Buffer supplemented with Complete protease inhibitor cocktail (Roche). Pierce™ Rapid Gold BCA Protein Assay Kit was

used to determine the protein concentrations (A53225, Thermo Scientific), and extracts were normalized for protein content. Whole-cell extracts were resolved by 10% sodium dodecyl sulfate (SDS) polyacrylamide gel electrophoresis and transferred onto polyvinylidene difluoride membrane (PVDF, Millipore). Primary antibodies including α/β-tubulin (2148S), Pan-actin (8456S), Bcl-2 (3498S), Bax (2772s), Caspase-9 (C9) (9508s), Cleaved caspase-9 (Asp353) (9509s), Cleaved-Caspase-3 (Asp175) (5A1E) (9664S), Caspase-3 (D3R6Y) (14220S), PARP (9542S) were purchased from Cell Signaling Technology (CST); GAPDH (7E4) (200306-7E4) was from ZEN BIO; horseradish peroxidase-conjugated secondary anti-mouse or anti-rabbit antibodies were purchased from Jackson Lab (115-035-003, 111-035-003). Detection was with the SuperSignal™ West Atto Ultimate Sensitivity Substrate (A38555, Thermo Scientific).

1.9. Endocytic uptake analysis

The 4T1 cells were seeded in a confocal dish at 5×10^4 cells/mL for 24 h and then treated with BP-PTX-Gd NPs in RPMI 1640 medium (equivalent to 0.2 μg Cy5.5/mL). The cells were incubated for 1 h, 2 h, 3 h, 4 h and 5 h, respectively. After PBS treatment, the cells were stained with Hoechst 33342 for 15 min in the dark. Finally, the cells were washed with PBS again and observed by a confocal laser scanning microscopy (CLSM) (PerkinElmer Operetta CLS). To investigate the intracellular delivery process of BP-PTX-Gd NPs, 4T1 cells were cultured as described above and subsequently incubated with the BP-PTX-Gd NPs for 1 h, 3 h, and 5 h, respectively. After staining with LysoTracker green and Hoechst 33342, cells were observed and photographed using CLSM.

1.10. Cell uptake inhibition experiment

To study the endocytosis mechanism of BP-PTX-Gd NPs, different inhibitors were

used to investigate the effect on the uptake of 4T1 cells. 4T1 cell suspension (2×10^5 cells/mL) was seeded in 6-well plates and incubated for 24 h. Subsequently, the original medium in each well was replaced with a fresh medium containing sodium azide (1 mg/mL), genistein (200 μg/mL), chlorpromazine (10 μg/mL) and nocodazole (10 μg/mL), respectively. After incubation for 30 min, the medium was removed, medium containing BP-PTX-Gd NPs (equivalent to 0.2 μg Cy5.5/mL) and inhibitor was added and incubated for 2 h (the concentration of the inhibitor in the medium was kept constant). At the same time, in order to investigate the effect of temperature on endocytosis, the cells were incubated with medium containing BP-PTX-Gd NPs (equivalent to 0.2 μg Cy5.5/mL) for 2 h at 4 °C. After the completion of the incubation, the medium was removed, and after washing three times with pre-cooled PBS, the cells of each treatment group were separately collected, and the fluorescence intensity of Cy5.5 in the different treatment groups was quantitatively detected by flow cytometry. Cells incubated at 37 °C in medium containing BP-PTX-Gd NPs and without inhibitor were used as controls and the fluorescence intensity from the control group was set to 100%.

1.11. Investigating the uptake in multicellular 4T1 tumor spheroids

To investigate the uptake of BP-PTX-Gd NPs by 4T1 tumor spheroids, 4T1 cells were seeded in 96-well U-bottom plates at a density of 1.5×10^3 cells per well. After 5 days of incubation, tumor spheroids were formed with a diameter of approximately 300 μm. Subsequently, the tumor spheroids were incubated with the BP-PTX-Gd NPs (equivalent to 0.2 μg Cy5.5/mL) for 1 h to 5 h, respectively. After washing with PBS, the uptake of BP-PTX-Gd NPs in the tumor spheroids at different incubation time points was observed by CLSM.

1.12. Fluorescence imaging of microtubules and microfilaments

For microtubule morphology analysis，4T1 cells were seeded in a confocal dish at 5×10^4 cells/mL for 24 h. Subsequently, 4T1 cells were treated with Taxol® and BP-PTX-Gd NPs in RPMI 1640 medium (equivalent to 0.5 μg PTX/mL) for 24 h, fixed with 4% paraformaldehyde, rinsed with PBS containing 0.1% Triton X-100. The cells were then incubated with the Tubulin-Tracker Red (diluted with PBS containing 3% bovine serum albumin (BSA) and 0.1% Triton X-100, 1:100) for 1 h at 37 °C, rinsed with PBS containing 0.1% Triton X-100, stained with 4',6-diamidino-2-phenylindole (DAPI) and imaged through CLSM.

For microfilaments morphology analysis, 4T1 cells were seeded in a confocal dish at 5×10^4 cells/mL for 24 h. Subsequently, cells were treated with Taxol® or BP-PTX-Gd NPs in RPMI 1640 medium (equivalent to 0.5 μg PTX/mL) for 24 h, fixed with 4% paraformaldehyde, rinsed with PBS containing 0.1% Triton X-100. The cells were then incubated with the Actin-Tracker Green (diluted with PBS containing 3%BSA and 0.1% Triton X-100, 1:100) for 1 h at 37 °C, rinsed with PBS containing 0.1% Triton X-100, stained with DAPI and imaged through CLSM.

To further analyze the changes of microtubules and microfilaments in the treated cells, we analyzed the contents of Pan-actin and α/β tubulin in the cells by Western Blot. Cells were incubated with Taxol® or NPs in the same way as described above.

1.13. In-vitro, in vivo and ex-vivo imaging

The relaxation rates of BP-PTX-Gd NPs and Gd-DTPA were measured on a 3T MRI scanner (Siemens Sonata). BP-PTX-Gd-NPs and Gd-DTPA were dissolved in PBS buffer (0.1 M) at different Gd(III) concentrations (0.10~0.60 mM). T_1-weighted images were acquired for all samples. PBS was used as a control. The method for calculating the T_1 relaxivity were described in a previous report.[6]

In vivo MR imaging experments were performed using tumor-bearing BALB/c

female mice. The mice (n = 5) were injected (i.v.) with BP-PTX-Gd NPs and Gd-DTPA, respectively, at a Gd(III) dose of 0.08 mmol Gd/kg. Mice were scanned at predetermined time intervals. MR contrast-enhanced images were obtained on a 3T MRI scanner, and the signal changes of the tumor were evaluated by semi-quantitative analysis as previously described.[6]

When tumors in BALB/c female mice reached around 150 mm^3, the mice were injected (i.v.) with BP-PTX-Gd NPs (equivalent to 1 mg Cy5.5/kg mice) to study their *in vivo* biodistribution. After administration, mice were euthanized at 1 h, 6 h, 12 h, 24 h, 48 h, 72 h, 96 h and 120 h for five mice. Subsequently, main organs and tumors of the mice were detached for fluorescence imaging and semi-quantitative analysis using the imaging system. The mice treated with saline were used as a control.

The 4T1 xenograft tumor bearing mice were randomly divided into two groups (n = 5) and injected intravenously with 200 μL of different formulations of Gd-DTPA or Gd-DTPA-loaded conjugates (equivalent 0.08 mmol Gd(III) /kg mice) via the tail vein, respectively. Mice were anesthetized and immobilized in the coil before being placed in the iso-center of the magnet. The contrast-enhanced imaging study was carried out on a 3T imaging system (Siemens Sonata Medical System, TE = 20 ms, TR= 500 ms, FOV = 40 ms, slice thickness = 1.0 mm, and flip angle = 90°).[7] The MRI images of each experiment group were acquired at the predetermined time point (pre-injection, 10 min, 0.5 h, 1 h, 4 h and 24 h post-injection). The relative enhancement of the signal-to-noise ratio (ΔSNR) was calculated as follows: $\Delta SNR = SI_{tumor} / SI_{water}$ (SI_{tumor} and SI_{water} were the signal intensity of tumor and water mold, respectively). In addition, the semi-quantitative analysis was applied to evaluate the signal changes by plotting the ΔSNR *versus* time.

1.14. Blood compatibility and biosafety analysis of BP-PTX-Gd NPs

Fresh blood used in the experiment was taken from healthy mice and stored in heparinized tubes. Blood compatibility evaluation was carried out in the same method as that from previous literature report.[8]

For hemolysis assays, isolated red blood cells were first obtained by centrifugation of 1 mL of fresh blood (1000 × g, 5 min). Subsequently, 30 μL of re-dispersed red blood cells were added to BP-PTX-Gd NPs containing PBS at different concentrations (1, 2 and 4 mg/mL, respectively). Under the same condition, red blood cells were added to distilled water and PBS (pH 7.4) respectively as a positive control and a negative control. After gentle shaking, each sample was incubated at room temperature for 4 h and then centrifuged at 1000 g for 5 min. Finally, the centrifuged samples were photographed, and the absorbance of each sample supernatant was recorded by a ultraviolet–visible (UV-vis) spectrophotometer at a wavelength of 540 nm. The percentage of hemolysis induced by BP-PTX-Gd NPs was calculated similarly as previous reports.[8] Three parallel groups were tested for each concentration.

20 μL of red blood cells were added to 100 uL of PBS containing BP-PTX-Gd NPs at different concentrations (1, 2 and 4 mg/mL, respectively). After a slight shock, the mixed solution was incubated for 15 min at 37 °C. After centrifuging to remove the supernatant, the cells were treated with 4% paraformaldehyde. The treated cells were resuspended in 0.5 mL PBS. Subsequently, 10 μL of the cells were loaded onto a glass slide, and dehydrated in a mixed solution of water and ethanol at different volumetric ratios (75%, 85%, 95%, and 100%, respectively). The sample was used for SEM observation after drying.

Blood routine and blood biochemical experiments were performed according to the previous literature. [9] Briefly, for blood chemistry tests, healthy female Balb/c mice

were randomly divided into 3 groups (n = 4). Then, BP-PTX-Gd NPs and Taxol® in saline at an equivalent dose of 10 mg PTX/kg mice were intravenously administered to mice every 4 days (four times in total). After 21 days, the mice were anesthetized and the eyeballs were removed, and blood was collected and used for blood biochemical tests. Mice treated with PBS were used as blank controls. Blood routine analysis was performed in the same manner as described above.

1.15. Pharmacokinetics studies

The pharmacokinetic *in vivo* behavior of BP-PTX-Gd NPs was analyzed by detecting the content of Gd(III) in the blood. Healthy female BALB/c mice (20 ± 2 g) were injected (i.v.) with Gd-DTPA and BP-PTX-Gd-NPs through the tail vein, respectively (Gd(III) dose of 0.08 mmol/kg, n = 5). At the scheduled time point ranging from 5 min to 96 h, 20 μL blood was collected from the fundus venous plexus of each mouse and placed in a centrifuge tube. The blood sample was thoroughly digested with concentrated HNO_3 and the liquid was evaporated under heating. The sample residue was reconstituted in distilled water, and the concentration of Gd(III) was detected by ICP-MS. PKSolver software was used to calculate the pharmacokinetic parameters.

In addition, to further analyze the pharmacokinetic behavior of BP-PTX-Gd NPs in vivo, we studied the abdominal vascular imaging of tumor-bearing mice by a 7.0 T small animal MRI scanner (Biospec 70/30USR, Bruker, Germany). Nine tumor bearing mice were randomly divided into three groups (n = 3). After anesthesia with isoflurane, mice in each experimental group were first pre-scanned. Subsequently, the mice were injected (i.v.) with Gd-DTPA and BP-PTX-Gd-NPs through the tail vein, respectively (Gd(III) dose of 0.08 mmol/kg), mice injected with saline were used as blank control. Abdominal blood vessel images of the mice were then obtained by

scanning at predetermined time points (10 min, 30 min, 90 min, 240 min and 480 min). Vascular imaging was performed using 3D fast imaging with steady-state precession (3D-FISP) sequence with the following parameters: TE = 2.6 ms, TR = 5.5 ms, Resolution = 0.12 × 0.12 × 0.35, Flip angle = 10°, and Scan time = 7 min. Images were reconstructed by Radiant DICOM software (v5.0.1).

1.16. Biodistribution analysis of the MRI agents

The 4T1 tumor-bearing mice were divided into two groups. BP-PTX-Gd NPs and Gd-DTPA were injected (i. v.) into tumor bearing mice, respectively, at a Gd(III) dose of 0.08 mmol Gd/kg. At 24 h post injection (five mice for each time point), after all mice were sacrificed, tumor and main organs of each mouse were dissected, weighed and recorded, and then digested in aqua regia. After they were completely digested under heating, each sample was reconstitution in distilled water, and ICP-MS was used to detected the Gd(III) concentration. For each sample, the Gd(III) content was calculated as the ratio of the percentage of the injected dose to the weight of each organ or tumor (%injected dose/gram, %ID g^{-1}). The biodistribution of MRI contrast agents in mice after 120 h post injection was studied in the same manner as above.

1.17. Study on tumor vascular permeability of BP-PTX-Gd NPs

Fluorescence confocal microscopy was used to investigate the penetration of nanoparticles from tumors into tumors. Because of its superficial appearance and large volume, subcutaneous tumors are suitable for real-time observation of the penetration of nanoparticles from blood vessels into tumor tissues. Subcutaneous xenografts 4T1 tumor model was established as described above.

When 4T1 xenograft tumor volumes reached 100 mm^3, mice were intravenously administrated with Cy5.5-labeled BP-PTX-Gd NPs (1.5 mg/kg of Cy5.5). After the mice were anesthetized, the skin on the surface of the tumor was uncovered, and the

fluorescence change of the tumor site was immediately observed using a fluorescence microscope. During the whole experiment, mice were anesthetized with isoflurane.

1.18. Antitumor effect studies in 4T1 tumor-bearing mice

When the tumors grew to a volume of about 100 mm^3, the mice were divided into three groups (n = 7): BP-PTX-Gd NPs, Taxol® and saline as a control. BP-PTX-Gd NPs and Taxol® in saline at an equivalent dose of 10 mg PTX/kg mice were intravenously administered to mice every 7 days three times. The body weight and tumor volume were recorded every 2 days until day 21. The relative tumor volume was calculated as the mean tumor volume on the measurement day/the mean tumor volume on the first day × 100%.

At the same time, accurate mouse tumor volume changes during treatment were monitored by MRI. Specifically, MR imaging study of the tumor imaging effects was performed using Bruker 7.0 T whole mouse MRI system. The T1 weighted SE sequence was: TR = 1500 ms, TE = 6.5 ms, slices =15, slices thickness= 0.7 mm, matrix = 256 × 256, voxel size = 0.137 × 0.137 × 0.7 mm3 and Fov = 35 mm × 35 mm. The tumor site magnetic resonance imaging in mice of different treatment groups was performed before administration, 4, 8, 12, 16, and 20 days after first administration. The volume fraction of the tumor site was measured by ITK-SNAP software (open source, version 3.6.0-RC1; http://www.itksnap.org/pmwiki/pmwiki.php). The phase image from MRI was selected for segmentation, with the region of interest covering the whole tumor.

In addition, to evaluate the in vivo antitumor effect of BP-PTX-Gd NPs by MRI, T1 map obtained by SE sequence was used to observe the T1 value changes of tumor sites after injection BP-PTX-Gd NPs/Taxol®/Saline (every 7 days, 4 times in total). Nine tumor-bearing female BALB/c mice (20 ± 2 g, 8-10 weeks) were randomly

divided into three groups (n = 3). After each administration, mice in the Taxol®-treated group and the saline-treated group were injected with a free Gd-DTPA solution (Gd concentration was the same as BP-PTX-Gd NPs, 0.054 mmol Gd/kg mice). Subsequently, three groups of mice were immediately analyzed by MRI. Specifically, MR imaging study of the tumor imaging effects was performed using a clinical Siemens 3.0 T MRI scanner equipped with a mouse coil. The T1 mapping sequence was: TR = 15-500 ms, TE = 2.0 ms, slices = 5, slices thickness= 2 mm, voxel size = 0.2 × 0.2 × 2 mm3, martix 256 × 256 and Fov = 160 × 160 mm. The tumor site magnetic resonance imaging was performed after administration 1, 7, 14, 21 day and T_1 value measurement of tumor sites using METALAB software.[10] The trend of the T_1 value of the tumor site during the treatment period of the experimental group was analyzed by $1/T_1$ versus time plot.

In order to obtain an improved anti-tumor effect, we examined the therapeutic effect of BP-PTX-Gd NPs on 4T1 tumor-bearing mice by referring to the above-mentioned treatment protocol and changing the frequency of administration to once every 4 days (four times in total). After all mice were sacrificed at the end of the experiment, tumor and major organs of each group were harvested. The tumor in each group was weighed to calculate tumor growth inhibition (TGI) using the formula: TGI (%) = $(1-W/W_0) \times 100\%$, where W and W_0 referred to the tumor weight of the treatment group and the control group, respectively. Major organs from each group were H&E stained and the tumor tissue was stained for immunohistochemistry analysis as previously reported (see Supporting Information).[28] Healthy mice treated with BP-PTX-Gd NPs were also treated as described above, and pathological changes in the major organs of the mice were observed by H&E staining at the end of treatment.

1.19. Immunohistochemistry analysis of CD31, Ki-67 and TUNEL assay.

The immunohistochemical evaluation was performed using the streptavidin-peroxidase method.[11] First, after de-paraffinaged and rehydrated, the paraffin-embedded tumor sections incubated with monoclonal anti-CD31 antibodies (1:200) (Beijing Biosynthesis Biotechnology Co., LTD) and monoclonal antibody against Ki-67 (1:200) (Beijing Biosynthesis Biotechnology Co., LTD) overnight at 4 °C， respectively. The biotinylated goat anti-rabbit antibodies were applied as secondary antibodies at 1 : 200 for 20 min at room temperature. Finally, the IHC images were captured by the Motic Images Advanced software (Motic China Group CO., LTD.), and the positive-stained integrated optical density (IOD) of the CD31 and ki-67 was scaled with each image by Image-Pro Plus 6.0 software (Media Cybernetics, Bethesda, MD). The tumor microvessel density (MVD) and ki-67-positive area/total area were obtained by calculating the ratio of CD31 to total area of each photograph and the ki-67 density in each photograph, respectively.

In situ terminal deoxynucleotidyl transferase-mediated UTP end labeling (TUNEL) assay was performed using an *in situ* cell death detection kit (Roche Molecular Biochemicals, Laval, Quebec, Canada) according to the manufacturer's instructions. An optical microscopy was used to observe the positive TUNEL staining and the ratio of the apoptotic cell number to the total tumor cell number in each microscope field was calculated as the apoptotic index.

1.20. Statistical analysis

Statistical analysis was carried out by student's t test and one-way ANOVA for comparison of two groups. Data were presented as mean ± SD. $p < 0.05$ and $p < 0.01$ referred to statistically significance and highly significance, respectively.

2. Results

Table S1. Characterization of the prepared branched pHPMA-PTX-Gd-Cy5.5 conjugate.

MW[a]	PDI	Gly[b]	Phe[b]	Leu[b]	Lys[b]	PTX[b]	Gd[b]	Cy5.5[b]	Size[c]	Size[d]	ζ[e]
186	2.30	2.45	2.86	2.16	1.64	5.36	6.5	0.7	62.6	50.0	-7.75

[a]The molecular weight (MW) was in kDa.

[b]The contents of amino acids, drug PTX, Gd and Cy5.5 contents were in weight percent (%).

[c]Size was confirmed by DLS as d.nm.

[d]Size was confirmed by SEM as d.nm.

[e]Zeta potential (ζ) was confirmed as mV.

Table S2. Degraded products after incubation of the branched pHPMA-PTX-Gd-Cy5.5 conjugate in McIlvaine's buffer with cathepsin B (2.8 μM, pH = 5.4) and PBS (pH 7.4) at 37 °C.

Conditions	0	2 h	6 h	12 h	18 h
Cathepsin B	186 kDa, PDI 2.30	132 kDa, PDI 2.50	68 kDa, PDI 1.80	25 kDa, PDI 1.20	25 kDa, PDI 1.22
PBS	186 kDa, PDI 2.30	185 kDa, PDI 2.30	184 kDa, PDI 2.33	183 kDa, PDI 2.36	182 kDa, PDI 2.42

Table S3. Pharmacokinetic parameters of BP-PTX-Gd NPs and Gd-DTPA by fitting the data to a two compartment model by PKSolver 2.0 software.

Parameters	Gd-DTPA	BP-PTX-Gd NPs
AUC(0-∞)(μg/mL·min)	2127.8 ± 319.9	39152.8 ± 7310.3
$T_{1/2}$(h)	0.27 ± 0.13	13.63 ± 1.42
Cl (L/min/kg)	0.118 ± 0.025	0.043 ± 0.0017
MRT(0–∞)(min)	22.89 ± 1.52	1106.0 ± 318.6

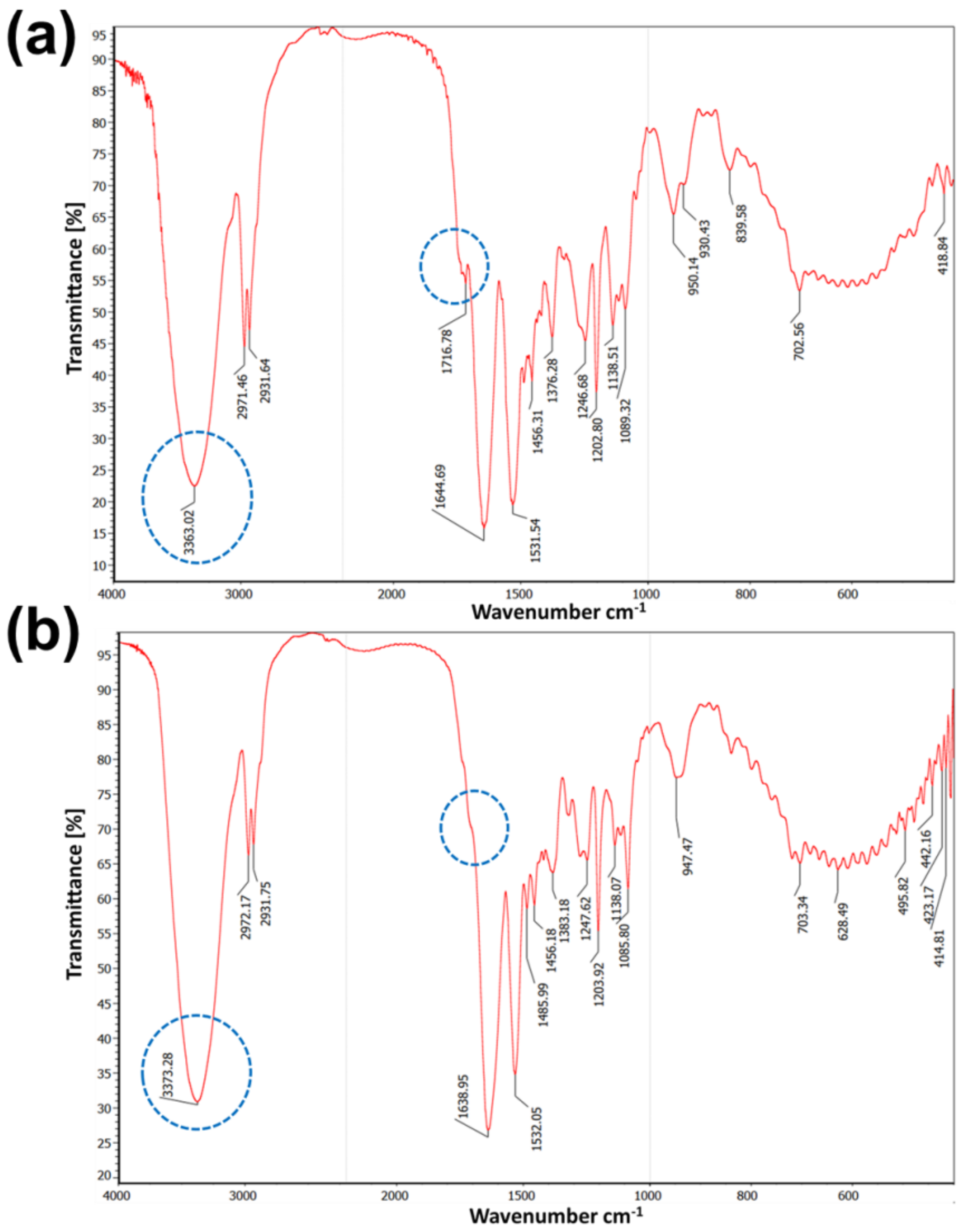

Figure S2. FTIR spectra of (a) polyHPMA-PTX-DOTA-Dithiopyridine and (b) polyHPMA-PTX-Gd-Cy5.5. The peak at 3363.02 cm^{-1} was the stretching vibration of hydroxyl group, and the peak at 1716.78 cm^{-1} was the stretching vibration of carbonyl group.

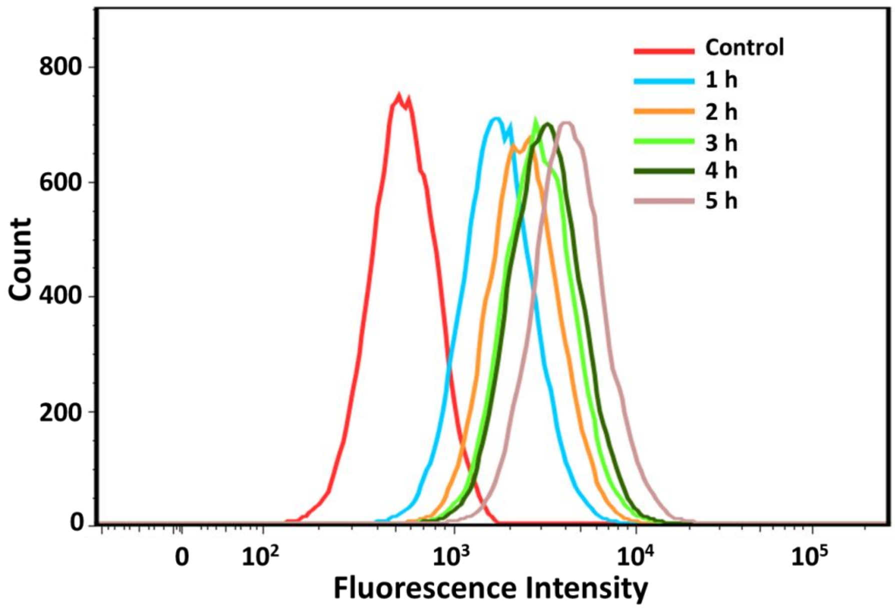

Figure S3. Quantitative fluorescence intensity of BP-PTX-Gd NPs that penetrate into 4T1 cells after incubation for 1 to 5 h.

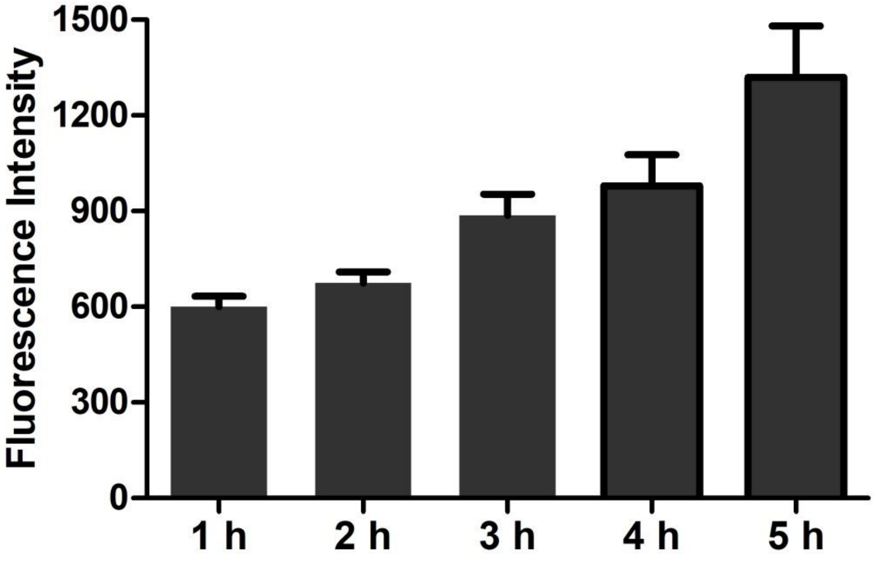

Figure S4. Quantitative fluorescence intensity of BP-PTX-Gd NPs that penetrate into 4T1 tumor spheroids after incubation for 1 to 5 h.

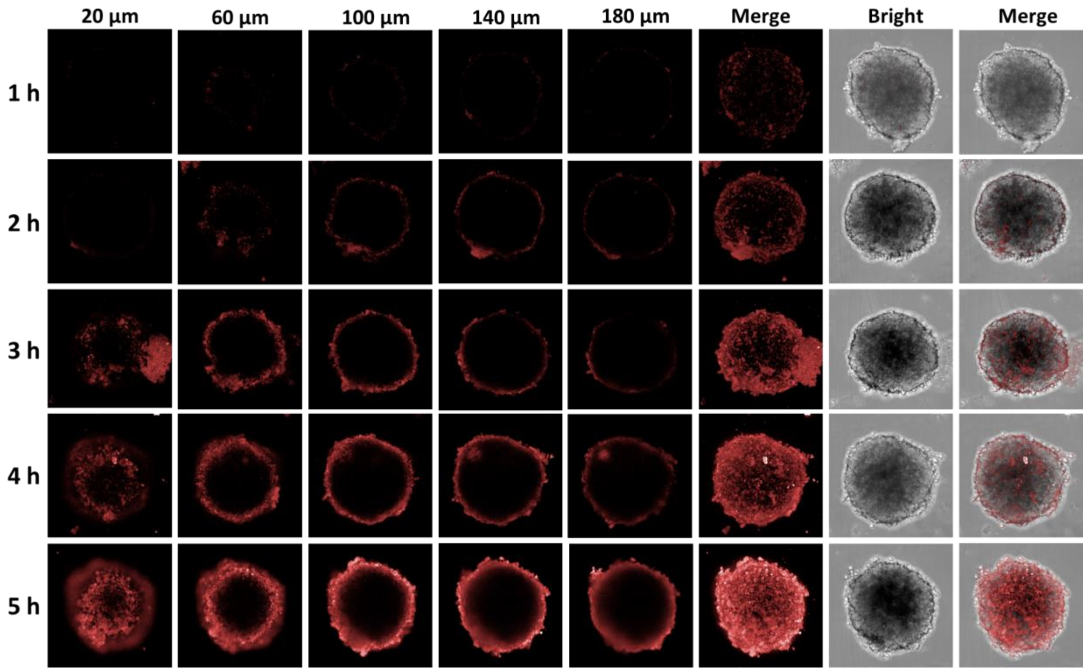

Figure S5. Tomographic scanning of 4T1 tumor spheroids from top to bottom after uptake of BP-PTX-Gd NPs for 1 to 5 h.

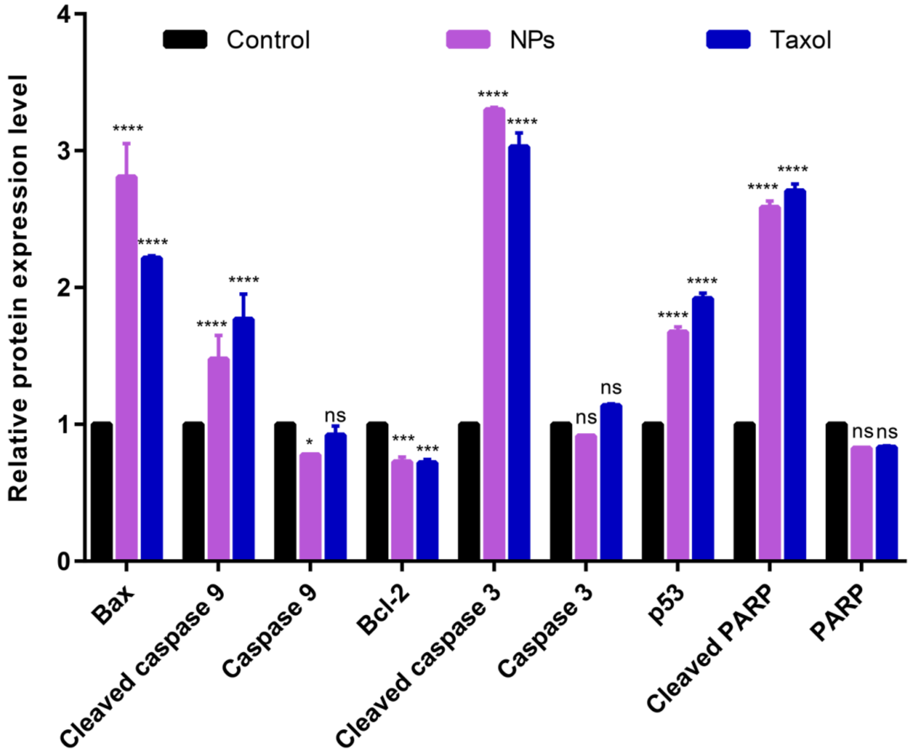

Figure S6. Quantification of relative expression levels of PARP, cleaved PARP, p53, caspase-3, cleaved caspase-3, Bcl-2, Bax, Caspase-9 and Cleaved Caspase-9. The blots were analyzed by densitometry and normalized to GAPDH. *P<0.05, **P<0.01, ****P<0.0001 compared to the control group (PBS), mean± SD (n=3).

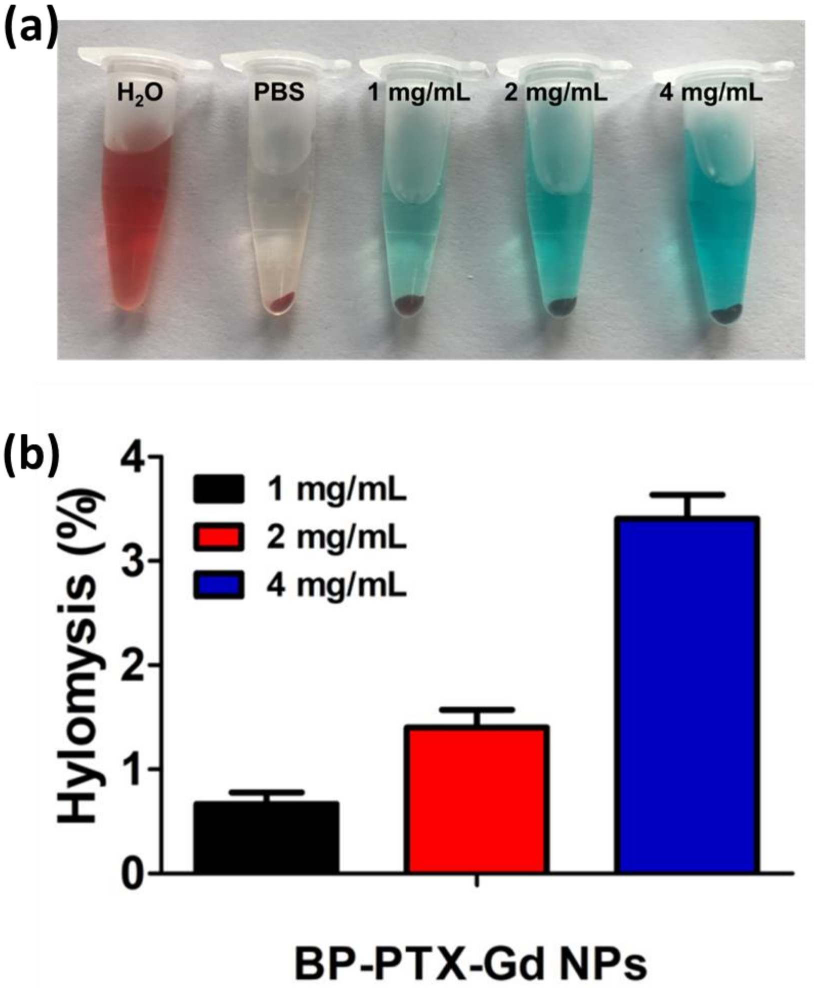

Figure S7. (a) Hemolysis of RBCs incubated with different concentrations of BP-PTX-Gd NPs, distilled water as a positive control and PBS as a negative control. (b) Quantitative analysis of the percentage of hemolysis of RBCs at different BP-PTX-Gd NPs concentrations.

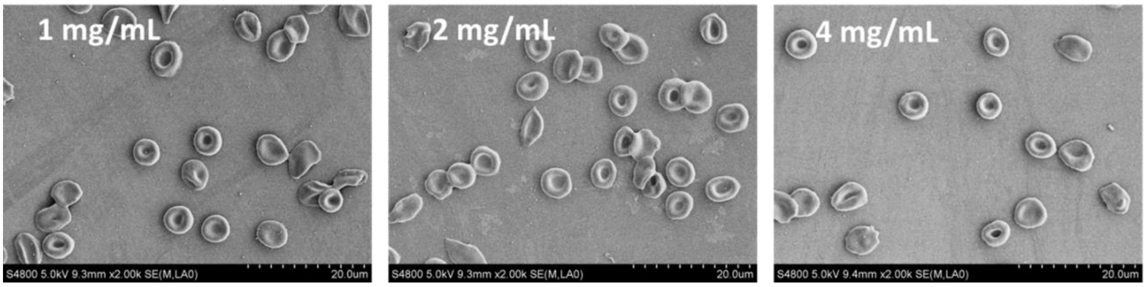

Figure S8. Effects of BP-PTX-Gd NPs at different concentrations on RBCs morphology and aggregation through SEM analysis.

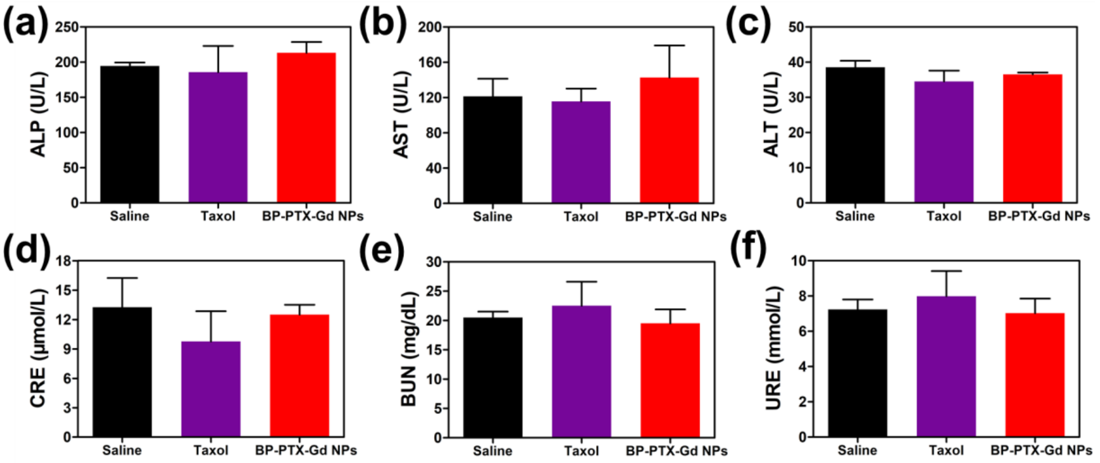

Figure S9. In vivo biosecurity assessment of BP-PTX-Gd NPs. Blood biochemistry test: (a) ALP, (b) AST, (c) ALT, (d) CRE, (e) BUN, (f) URE.

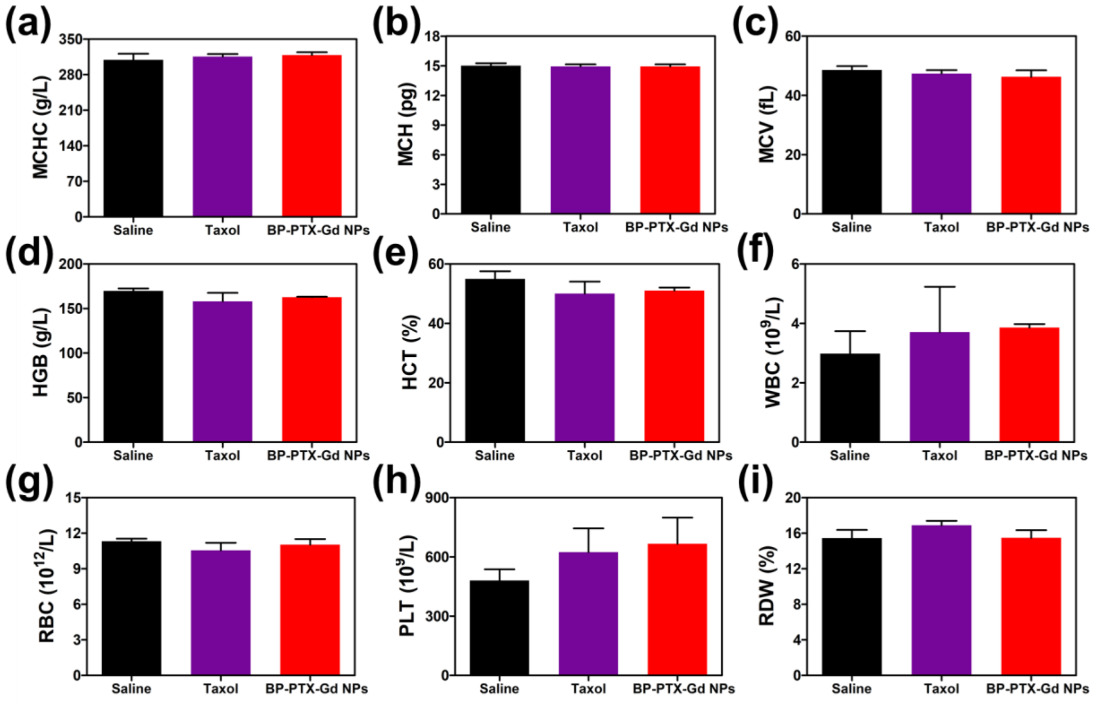

Figure S10. In vivo biosecurity assessment of BP-PTX-Gd NPs. Routine blood analysis: (a) MCHC; (b) MCH; (c) MCV; (d) HGB; (e) HCT; (f) WBC; (g) RBC; (h) PLT; (i) RDW.

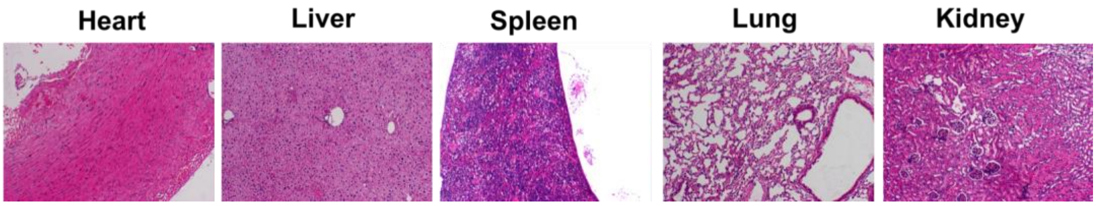

Figure S11. H&E stained histological sections of major organs of normal mice on 21 d after treatments with BP-PTX-Gd NPs.

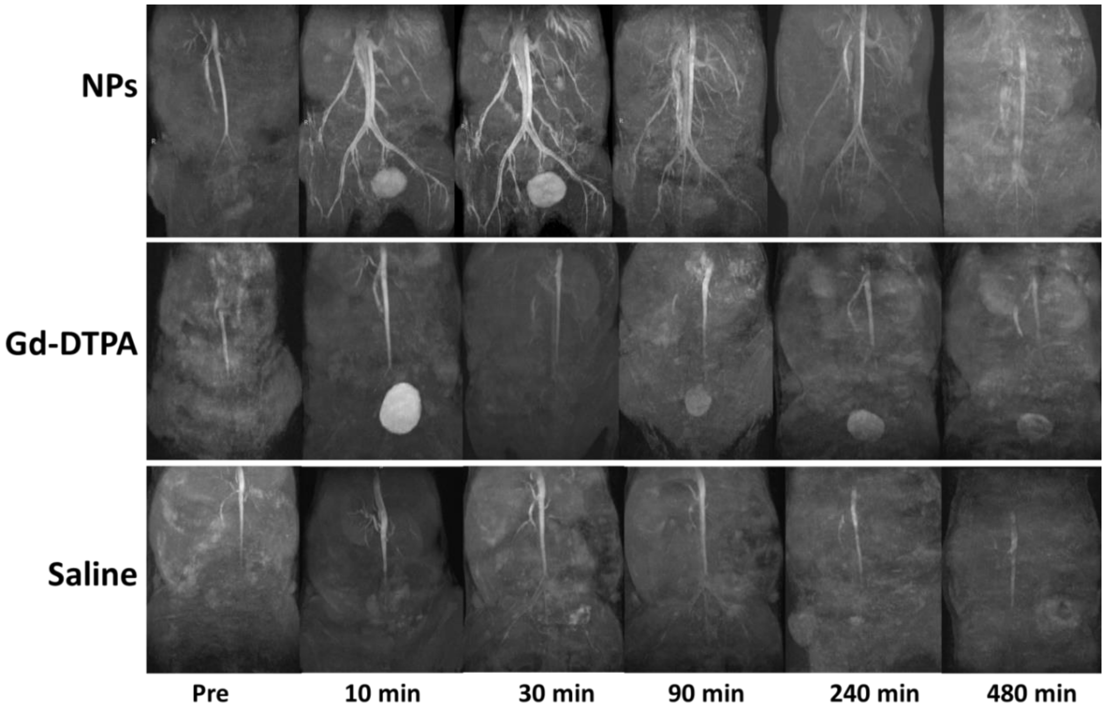

Figure S12. Abdominal vascular imaging of mice in different groups at different time points after injection of BP-PTX-Gd NPs, Gd-DTPA or saline.

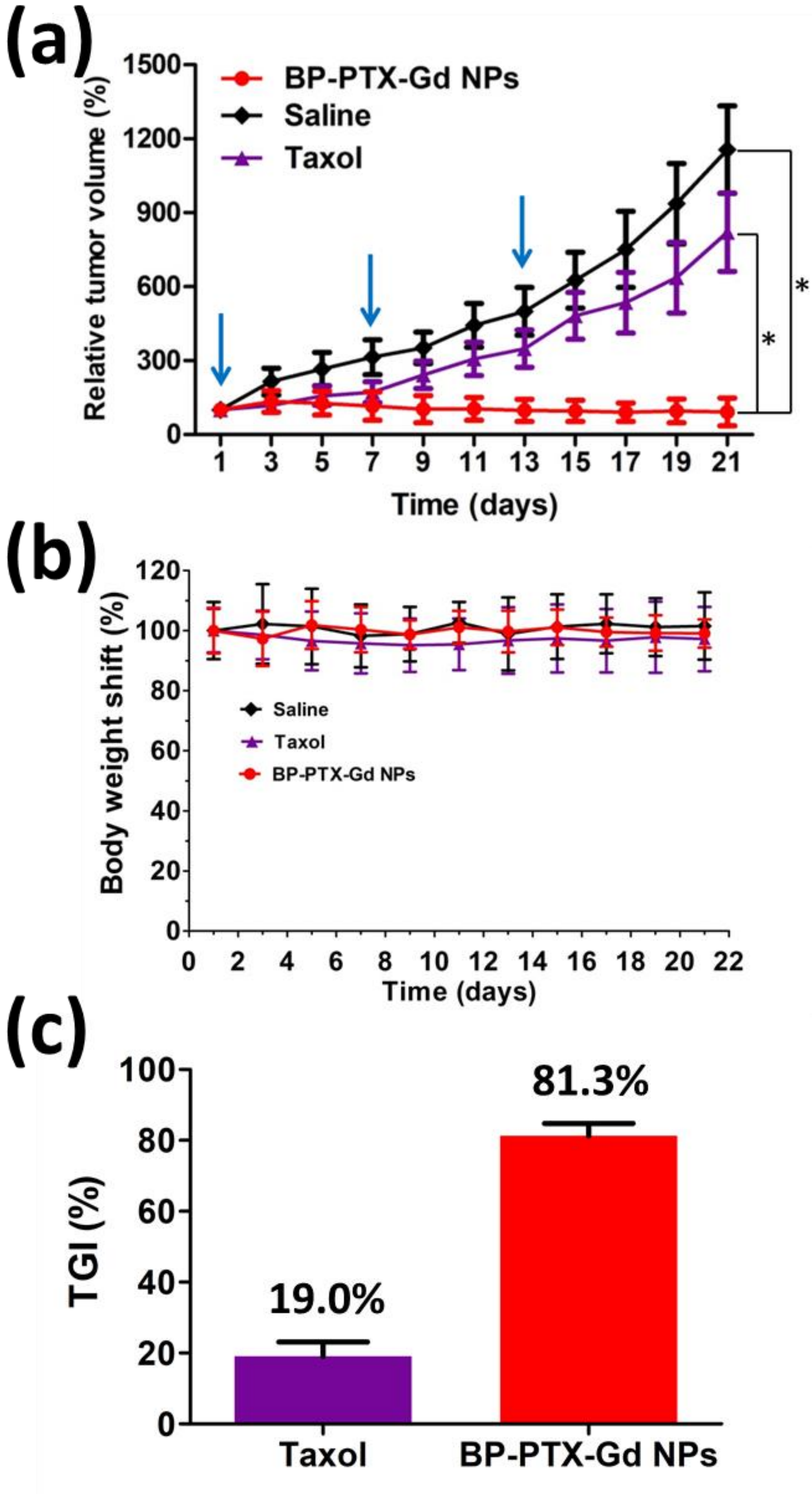

Figure S13. (a) Tumor volume changes in 4T1 tumor-bearing mice treated with saline, Taxol® and BP-PTX-Gd NPs by intravenous injection (10 mg PTX/kg mice, mean ± SD, n = 7, *p < 0.001). Arrows represent the point in time of administration. (b) Mice average body weight shifts post-injections of BP-PTX-Gd NPs, Taxol® and saline up to day 21. (c) Tumor growth inhibition (TGI, %) after administered with saline, Taxol® and BP-PTX-Gd NPs for 21 days.

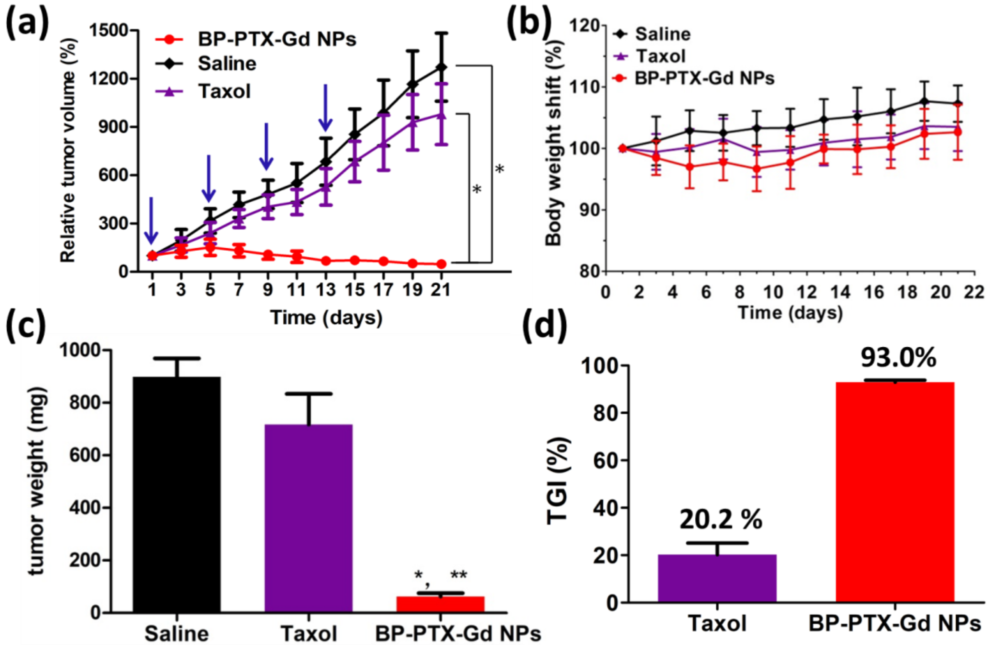

Figure S14. (a) Tumor volume changes in 4T1 tumor-bearing mice treated with saline, Taxol® and BP-PTX-Gd NPs by intravenous injection (10 mg PTX /kg mice, mean ± SD, n = 7, *p < 0.001). Arrows represent the point in time of administration. (b) Mice average body weight shifts post-injections of BP-PTX-Gd NPs, Taxol® and saline up to day 21. (c) Tumor weight of mice treated with Saline, Taxol or BP-PTX-Gd NPs after 21 days of treatment. (*p < 0.001 vs saline, **p < 0.001 vs Taxol®). (d) Tumor growth inhibition (TGI, %) after administered with saline, Taxol® and BP-PTX-Gd NPs for 21 days.

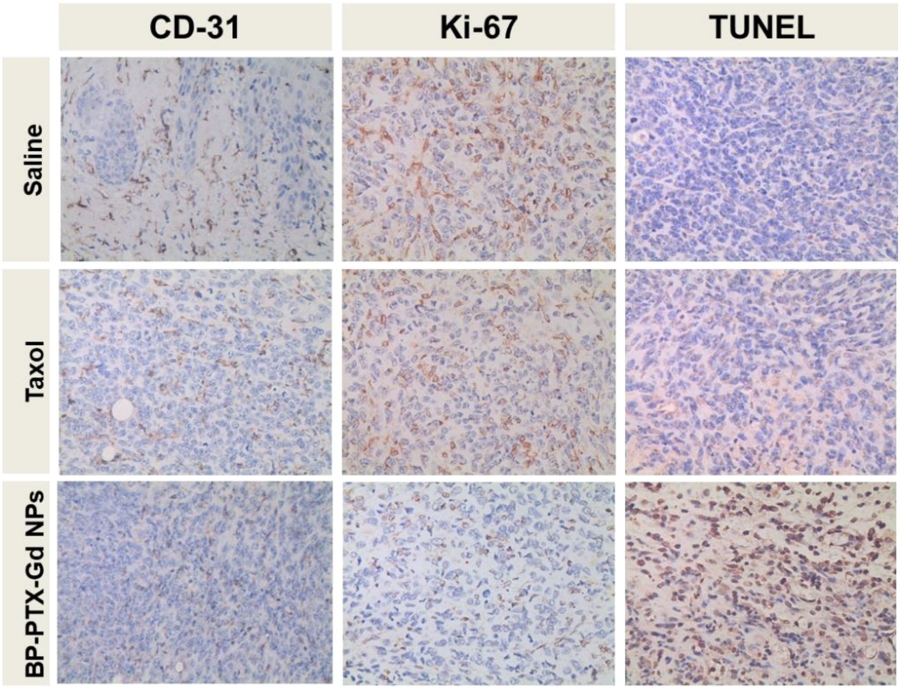

Figure S15. Immunohistochemical analysis of CD31, Ki-67, and TUNEL assays for 4T1 tumors. Brown for positive staining.

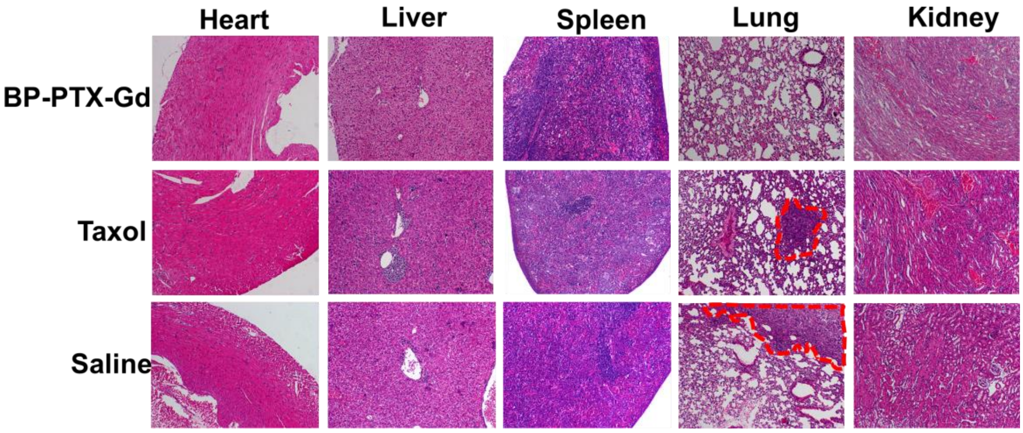

Figure S16. H&E sections of major organs in mice treated with saline, Taxol and BP-PTX-Gd NPs for 21 days.

REFERENCES

[1] R. Zhang, J. Yang, M. Sima, Y. Zhou, J. Kopecek, *Proc. Natl. Acad. Sci. USA* **2014**, 111, 12181.

[2] M. L. Tang, M. L. Zhou, Y. A. Huang, J. J. Zhong, Z. Zhou, K. Luo, *Polym. Chem.* **2017**, 8, 2370.

[3] L. Sun, X. Li, X. L. Wei, Q. Luo, P. J. Guan, M. Wu, H. Y. Zhu, K. Luo, Q. Y. Gong, *ACS Appl. Mater. Interfaces* **2016**, 8, 10499.

[4] R. Zhang, K. Luo, J. Yang, M. Sima, Y. Sun, M. M. Janat-Amsbury, J. Kopecek, *J. Control. Release* **2013**, 166, 66.

[5] X. L. Wei, Q. Luo, L. Sun, X. Li, H. Y. Zhu, P. J. Guan, M. Wu, K. Luo, Q. Y. Gong, *ACS Appl. Mater. Interfaces* **2016**, 8, 11765.

[6] X. Li, L. Sun, X. Wei, Q. Luo, H. Cai, X. Xiao, H. Zhu, K. Luo, *J. Mater. Chem. B* **2017**, 5, 2763.

[7] C. H. Guo, L. Sun, W. C. She, N. Li, L. Jiang, K. Luo, Q. Y. Gong, Z. W. Gu, *Polym. Chem.* **2016**, 7, 2531.

[8] C. H. Guo, L. Sun, H. Cai, Z. Y. Duan, S. Y. Zhang, Q. Y. Gong, K. Luo, Z. W. Gu, *ACS Appl. Mater. Interfaces* **2017**, 9, 23508.

[9] a) W. Yang, W. Guo, W. Le, G. Lv, F. Zhang, L. Shi, X. Wang, J. Wang, S. Wang, J. Chang, B. Zhang, *ACS Nano* **2016**, 10, 10245; b) X. Lu, Y. Zhu, R. Bai, Z. Wu, W. Qian, L. Yang, R. Cai, H. Yan, T. Li, V. Pandey, Y. Liu, P. E. Lobie, C. Chen, T. Zhu, *Nat. Nanotechnol.* **2019**, 14, 719; c) Z. Wang, Y. Ju, Z. Ali, H. Yin, F. Sheng, J. Lin, B. Wang, Y. Hou, *Nat. Commun.* **2019**, 10, 4418.

[10] D. Laurent, J. Wasvary, E. O'Byrne, M. Rudin, *Magnet. Reson. Med.* **2003**, 50, 541.

[11] N. Li, N. Li, Q. Yi, K. Luo, C. Guo, D. Pan, Z. Gu, *Biomaterials* **2014**, 35, 9529.
